# Prevalence and Diversity Analysis of Candidate Prophages to Provide An Understanding on Their Roles in *Bacillus Thuringiensis*

**DOI:** 10.3390/v11040388

**Published:** 2019-04-25

**Authors:** Yajuan Fu, Yan Wu, Yihui Yuan, Meiying Gao

**Affiliations:** 1 Wuhan Institute of Virology, Chinese Academy of Sciences, Wuhan 430071, China; fuyajuann@163.com (Y.F.); wuyan_81@126.com (Y.W.); 2 University of Chinese Academy of Sciences, Beijing 100039, China; 3 Present address: State Key Laboratory of Marine Resource Utilization in South China Sea, Hainan University, Haikou 571158, China

**Keywords:** *Bacillus thuringiensis*, prophage, phage, superinfection exclusion, antibiotic resistance gene

## Abstract

*Bacillus thuringiensis* (Bt) is widely used in producing biological insecticides. Phage contaminations during Bt fermentation can cause severe losses of yields. Lots of strategies have been engaged to control extrinsic phage contamination during Bt fermentation, but their effectiveness is low. In this study, the candidate endogenous prophages (prophages) in 61 Bt chromosomes that had been deposited in GenBank database were analyzed. The results revealed that all chromosomes contained prophage regions, and 398 candidate prophage regions were predicted, including 135 putative complete prophages and 263 incomplete prophage regions. These putative complete prophages showed highly diverse genetic backgrounds. The inducibility of the prophages of ten Bt strains (4AJ1, 4BD1, HD-1, HD-29, HD-73, HD-521, BMB171, 4CC1, CT-43, and HD-1011) was tested, and the results showed that seven of the ten strains’ prophages were inducible. These induced phages belonged to the *Siphoviridae* family and exhibited a broad host spectrum against the non-original strains. The culture supernatants of the two strains (BMB171, 4CC1) could lyse Bt cells, but no virions were observed, which was speculated to be caused by lysin. The functional analysis of the putative complete prophage proteins indicated that some proteins, such as antibiotic resistance-associated proteins and restriction endonucleases, might increase the fitness of the Bt strains to different environments. The findings of this study provided understanding on the high prevalence and diversity of Bt prophages, as well as pointed out the role of prophages in the life cycle of Bt.

## 1. Introduction

*Bacillus thuringiensis* (Bt) is a ubiquitous Gram-positive, spore-forming bacterium that produces parasporal crystals at the end of its life cycle. Due to the insecticidal ability of the parasporal crystal proteins, Bt is widely used in producing bio-pesticides [1,2]. However, during Bt fermentation, the outbreak of phage contamination always causes severe losses on the yields of crystal and spore, which are the main active components against insect pests. The phage contamination usually results in losses of 15 to 30% of the total batches, sometimes up to 50 to 80%. At worst, the phage contamination may cause a failure in fermentation [3]. 

Though many strategies—such as screening phage-resistant Bt strains, treating the seed broth with a high temperature (≥85 °C), and strengthening the air filtration system—have been employed to control phage contamination outside bacterial cells, it is still a problem that interferes with Bt fermentation. For the purpose of understanding Bt phage diversity and providing instructions for constructing phage-resistant genetically engineered Bt strains, many Bt phages have been isolated and their interactions with the host bacteria have been characterized [4,5,6,7]. Temperate phages integrated their DNA into host chromosomes, and the process of replication with the host cell is called the lysogenic cycle. Under particular circumstances, temperate phages may be excised from the bacterial genome and enter lytic lifecycles, accompanied with bacterial lysis [8]. In addition, some temperate phages, such as P1, N15, and LE1, exist as circular or linear plasmids (the latter of them are called telomeres phages) [9,10,11], and prophages refer to all the virion DNA in the host genomes. The temperate phages harbor diverse mechanisms for determining lytic or lysogenic growth, such as the CI and Cro regulators in phage λ [12,13,14], the primary immunity region *immB* in telomere phages [15], and the site-specific recombination in mycobacteriophages [16]. The Bt temperate phages have been studied since the 1960s, and more than 83% of Bt strains have been found to contain temperate phages [17,18]. After the discovery of the first temperate phage, the features of several Bt temperate phages, such as Tm2, MZTP02, and BMBtp2 phages, were well analyzed, and these phages were mainly induced from Bt strains using mitomycin C [3,19,20,21,22]. In 2013, Jong analyzed the existence of temperate bacteriophages in Bt type strains [23]. The results showed that the prophages in 22 Bt type strains could spontaneously enter the lytic life cycle.

Temperate phages are common, and bacterial genomes are full of integrated prophages [24]. The investigation of prophages in 482 pneumococcal strains showed that every genome contained prophages, and some persisted for a long time without changes at the nucleotide sequence level [25]. In *Desulfovibrio*, 128 prophage-like elements were screened in 46 of the 47 analyzed genomes, and 53 were identified as complete prophages [26]. *Escherichia coli* O157 possesses 18 prophages, but most of them contain genetic defects and many of the defective prophages could be induced and released from the host cell [27]. Though several studies on other bacterial prophages and Bt temperate phages have been conducted, there is no systematic investigation on the prevalence and diversity of Bt prophages. Using multiple completed and available genomic sequences of Bt, it is possible to analyze the prevalence and diversity of Bt prophages. In this study, we predicted and analyzed the candidate prophage regions in 61 Bt chromosomes which were deposited in NCBI. Ten strains containing putative complete prophages were induced by mitomycin C, and the features of the inducible prophages were determined. The findings of this study provided a systematic understanding on the prophage diversity of Bt and might offer guidance for screening and constructing Bt strains that can avoid phage contamination during fermentation. The findings of this study also shed light on the roles of prophages in the Bt life cycle.

## 2. Materials and Methods

### 2.1. Collection of the Bt Genome Dataset and Prediction of Prophage Regions

The complete genome sequences or chromosomes of 61 Bt strains had been deposited in the GenBank database (https://www.ncbi.nlm.nih.gov/genome/genomes/486) before 30 December 2018. These Bt chromosome sequences were collected (Table S1), and the candidate prophage regions in each genome were predicted by using PHASTER [28]. The putative complete prophages were identified as previously reported [26]. In summary, we used PHASTER for the initial integrase and attL/attR prediction and redefined the genome boundaries according to the integrase position with the last phage-related gene. Next, we verified the essential structure genes using PSI-BLAST (https://www.ebi.ac.uk/seqdb/confluence/display/THD/PSI-BLAST) and VirFam (http://biodev.cea.fr/virfam/). The prophages containing all structural genes were regarded as putative complete prophages, and their sequences were extracted from the strain genomes and used for further bioinformatic analysis.

### 2.2. Bioinformatic Analysis of Prophage Sequences

In this study, we mainly focused on the putative complete prophages in Bt strains. The distance between the sequences of 135 putative complete prophage genomes was calculated by using MegAlign [29], and the phylogenetic tree was subsequently constructed based on the distance between each prophage in MEGA with the neighbor-joining method with a bootstrap of 1000. The amino acid sequences of the major capsid proteins and the large terminase subunits of the prophage regions were also used for the phylogenetic analysis of the putative complete prophages. The multiple sequence alignments of amino acid sequences were performed using the ClustalW algorithm with default parameters in MEGA 6.0, and the phylogenetic tree was constructed using the neighbor-joining method with a bootstrap of 1000 [30]. The phylogenetic trees of putative complete prophage genomes, the major capsid proteins, and the large terminase subunits were all visualized using iTOL (http://itol.embl.de/) [31]. The dot plot analysis of nucleotide sequences between prophages and phages infecting *Bacillus* strains was conducted using Gepard [32]. The comparative genomic analysis was carried out using Mauve [33]. The alignments of different antibiotic resistance genes with the reference genes were conducted with DNAman (https://www.lynnon.com/). The proteins encoded by the prophage regions were predicted by using Genmarks [34], and the COG (Cluster of Orthologous Groups of proteins) classification of the proteins was performed using WebMGA (http://weizhong-lab.ucsd.edu/metagenomic-analysis/server/cog/) [35].

### 2.3. Induction and Characteristic of Prophages from Genome-Sequenced Bt Strains 

The Bt strains (*B. thuringiensis* serovar. *jinghongiensis* YGd22-03, *guiyangiensis* KK31-01, *rongseni* Scg04-02, *pingluonsis* NXP15-04, *zhaodongensis* HZ39-04, *sinensis* YK30-04 and *wuhaniesis* 140) were identified and stored by our laboratory [36]. The BMB171 strain was provided by Dr. Ming Sun (State Key Laboratory of Agricultural Microbiology, Huazhong Agricultural University, Wuhan, China). The other Bt type strains were kindly provided by the Institute Pasteur (France). The 10 strains for phage induction assay were cultured in Luria–Bertani (LB) broth at 30 °C for 12 h, with moderate shaking. Then, they were transferred to another conical flask for 4 h, and mitomycin C was added into the cultures to a final concentration of 0.5 g/mL and cultivated for another 6 h. The induced cultures were collected via centrifugation at 10,000× *g* for 30 min, and the supernatants were collected and filtered through a filter with a pore size of 0.22 μm. The filtrate was incubated with 10% polyethylene glycol (PEG) 8000 and 0.5 M NaCl overnight at 4 °C [37], followed by centrifuging and the deposit was resuspended and filtered. The growth of the strains induced by using mitomycin C was monitored by measuring the optical density at 600 nm (OD_600_) at intervals of 1 h. The strains without induction were used as controls.

The infective abilities of the induced phages in the supernatants were determined by using the double agar overlay assay method, as described before, with some modifications [38]. In summary, 200 μL exponential growth strains were mixed with 5 mL semisolid medium, then poured onto a solid medium plate as an overlay. After solidification of the upper medium, 3 μL of the re-filtered culture supernatants of the 10 Bt strains were spotted onto the upper semisolid medium, and the formation of lysis zones was observed after incubation at 30 °C overnight. The assay was conducted in triplicate, and the three batches of induced supernatants were used for host spectrum determination. This was done at least three times to obtain comprehensive information. The phage particles in the induced supernatants were deposited onto copper grids that were carbon-coated with Formvar film, negatively stained with 2% phosphotungstic acid solution (pH 7.0), and then observed with transmission electron microscopy (TEM, H-7000FA, HITACHI, Tokyo, Japan) at an acceleration voltage of 100 kV.

The Bt strains, in plates which contain the most large and clear lysis zones, were used for phages purification. The HD-29 strain was used for the purification of induced phage from HD73 strain. The exponential growth strains (200 μL) were mixed with gradient diluted supernatants (200 µL), and then poured onto a solid medium plate as an overlay overnight. The plaques formed on the plates were used for phage purification using the method previously reported [22]. Simply, the amplified cultured phages were centrifuged with sucrose density gradient ultracentrifugation at 38,000× *g*/min for 2 h in a BeckmanCoulter Optima L-100 K ultracentrifuge [39]. After centrifugation, the phage band was collected, and the phages were dialyzed. The genomic DNA of the phage was extracted according to the methods previously described [25]. The genome of the phage was sequenced using an Illumina Hiseq 2500 (Illumina, San Diego, CA, USA) and de novo assembled into contigs with a SPAdes 3.5.0 assembler. Gaps between contigs of the phage genome were filled using primer walking to create complete genome sequences. The open reading frames (ORFs) were predicted via RAST annotation server (http://rast.nmpdr.org/rast.cgi) [40] and GeneMark (http://topaz.gatech.edu/GeneMark/gmhmmp.cgi) [41], and the protein function were further identified by using the Pfam 32.0 database (http://pfam.xfam.org/search/sequence) [42] and HHpred (https://toolkit.tuebingen.mpg.de/#/tools/hhpred) [43]. The similarity with the known phages was determined using PASC (https://www.ncbi.nlm.nih.gov/sutils/pasc/viridty.cgi?cmdresult=main&id=428) [44]. The phylogenetic analysis of the phage phiHD73 was performed by using MEGA 6.0 with the neighbor-joining method and bootstrap analysis (1000 replicates) [18].

### 2.4. In-Gel Lytic Activity Assay

The bacterial cultures induced by using mitomycin C were collected by centrifugation and subsequently filtered through a 0.22 μm filter. The filtrates were then concentrated by incubation with PEG 8000 and NaCl, followed by being centrifuged and re-filtered. The concentrated and re-filtered supernatants were further separated using SDS-PAGE containing 0.4% of the exponential growth and autoclaved host cells. The strains without mitomycin were used as controls. After separation, the gel was rinsed four times with 2.5% Triton X-100 and incubated in a renaturation buffer (50 mM 2-morpholinoethanesulfonic acid, 1% Triton X-100, 1 mM MgCl_2_, 1 mM CaCl_2_, pH 6.5) overnight at 37 °C, as previously described [45]. After that, the gel was rinsed with distilled water and stained with 1% methylene blue in 0.01% KOH. The lytic band was observed after the gel was rinsed with distilled water.

## 3. Results

### 3.1. High Prevalence of Prophage Sequences in Bt Genomes

**Figure 1:**
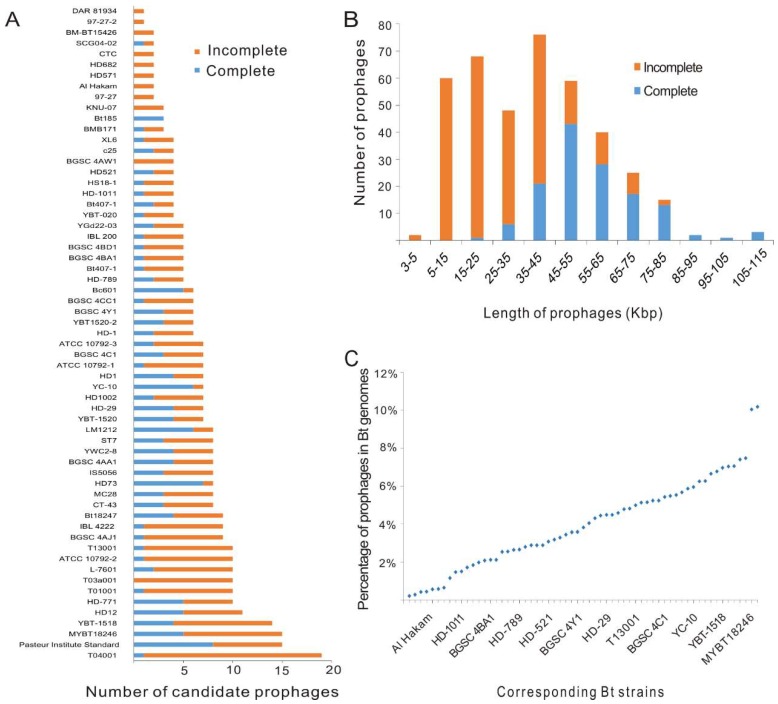
Characterization of candidate prophage regions identified in 61 Bt genomes. (**A**) The number of candidate prophage regions in each genome. (**B**) Sequence length distribution of all 398 candidate prophage regions identified in Bt genomes. (**C**) The percentage of the total genome size of all prophage regions from the same Bt genome in the corresponding strain.

The chromosome sequences of 61 Bt strains were used for prophage prediction, and all strains were found to contain candidate prophage regions (Table S1). In total, 398 candidate prophage regions were predicted, including 135 putative complete prophages and 263 incomplete prophages. The average number of prophage regions in each strain was about 6.52 (Table S2). The putative complete prophages, which contain the essential genes of phages and most likely can be induced from bacteria, were found in 50 (81.9%) Bt strains. The average number of putative complete prophages in all strains tested in this study was about 2.21. Among the 61 Bt genomes, most genomes were found to contain 5 to 10 prophage regions, while 5 genomes contained more than 10 prophage regions (Figure 1A). The length of putative complete prophage sequences ranged from 23–110 kbp (Figure 1B). The genome of the T04001 strain contained the most prophage regions, which included 19 prophage regions and took up to 7.40% of the bacterial chromosome (Table S1; Figure 1C). The prophage regions in the genome of the Pasteur Institute Standard strain reached up to 699 kbp and comprised 10.18% of the bacterial genome. The genome of the HD73 strain had the most putative complete prophages. 

### 3.2. Diversity and Phylogenetic Relationship Analysis of Putative Complete Prophages from Bt Genomes

**Figure 2:**
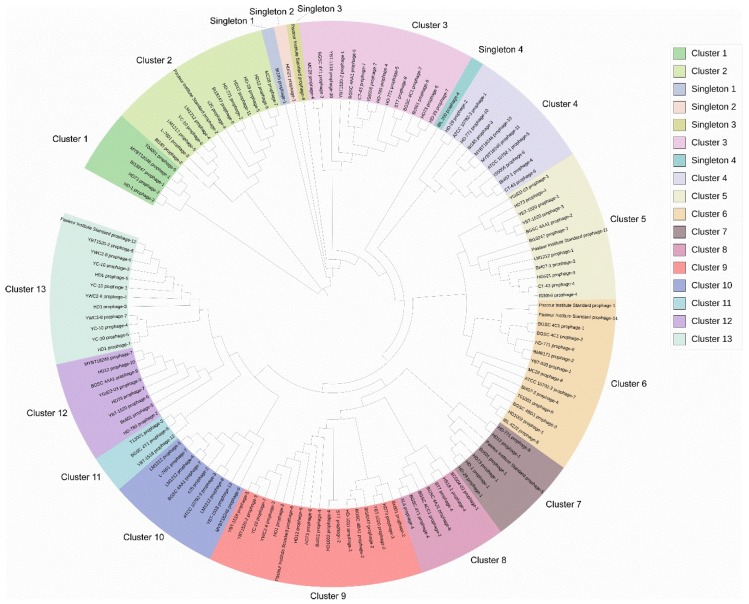
Phylogenetic analysis of the putative complete prophages in Bt genomes. The DNA sequences of 135 putative complete prophages were extracted from the 61 bacterial chromosomes and used for phylogenetic tree construction. The sequence distances of these prophages were calculated using MegAlign, and the phylogenetic trees were subsequently constructed. This was followed by visualization with iTOL.

The DNA sequences of 135 putative complete Bt prophages were used to analyze the phylogenetic relationship of the prophages. The result showed that these putative complete prophages could be classified into 13 clusters and 4 singletons (Figure 2). The prophages from the same Bt strain had a wide distribution, which corresponded with the result that the prophages in the same strain showed few similarities. The phylogenetic trees constructed based on the amino acid sequences of the major capsid proteins and the terminase large subunit proteins divided the prophages into two clades, and the prophages in each clade showed a similar evolutionary relationship with the phylogenetic tree based on genome sequence distance (Figures S1 and S2).

**Figure 3:**
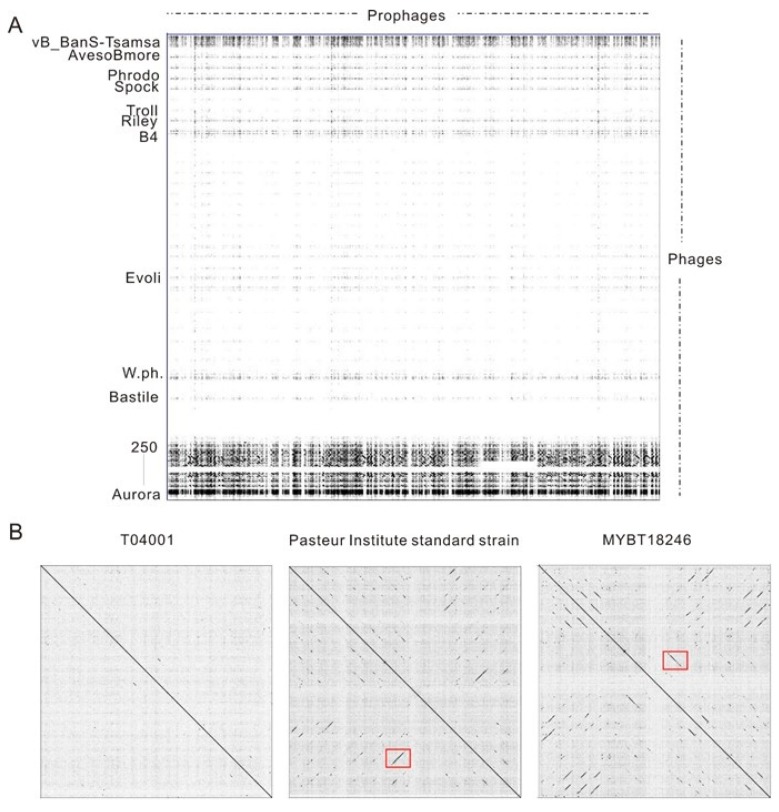
Pairwise comparison of prophages identified from Bt genomes using Gepard. (**A**) Comparisons of 135 putative complete Bt prophage sequences, with phages infecting *Bacillus cereus* group strains. The horizontal direction indicates the sequences of prophages, and the vertical direction indicates the phages sequences. Partial phages that the genome showed similarities with prophages regions were shown. The genome sequences of the 67 phages that infected the *B. cereus* group strains were collected from GenBank and ranked by the length of the phage genomes. This is listed in Table S4. (**B**) Pairwise comparison of all candidate prophage regions identified from the same strain. The co-linearity regions of the two red boxes mark structural proteins.

The similarities of the prophage regions to existing phage genomes were analyzed (Figure 3A, Table S2). The results indicated that the prophages exhibited high diversity. Of the 398 candidate Bt prophage regions, 41 prophages (including 13 putative complete prophages and 28 incomplete prophages) showed a high similarity (similarity higher than 50%) to the phage genomes. Also, 25.6% of the Bt prophages were similar to the *Bacillus* phages, with similarity lower than 10%. The DNA sequences of the prophages were mainly similar to temperate phages, such as Wβ-group phages, including phage BtCS33, phIS3501, phiCM3, Wβ, and some other Bt temperate phages, such as phage vB_BtS_BMBtp3, BMBtp1, vB_BtS_BMBtp16, and phi4B1 (Table S2). Four putative complete prophages were found to possess novel DNA sequences and showed low similarities with existing phage genomes, including LM1212 prophage-6, HD1 prophage-7, YGd22-03 prophage-1, and c25 prophage-4. Pairwise comparison analysis of the prophages revealed that the prophages in the same strain usually exhibited extremely low similarities (Figure 3B), and the co-linearity region mainly encoded structural proteins. The regions indicated by the two red boxes encode phage tail tape measure protein (100% coverage and 89.15% identity with *Bacillus* phage BtCS33) and major capsid protein (95% coverage and 92.22% identity with *Bacillus* phage phiCM3), respectively. 

### 3.3. Bioinformatic Analysis of Proteins Encoded by Bt Prophages

**Figure 4:**
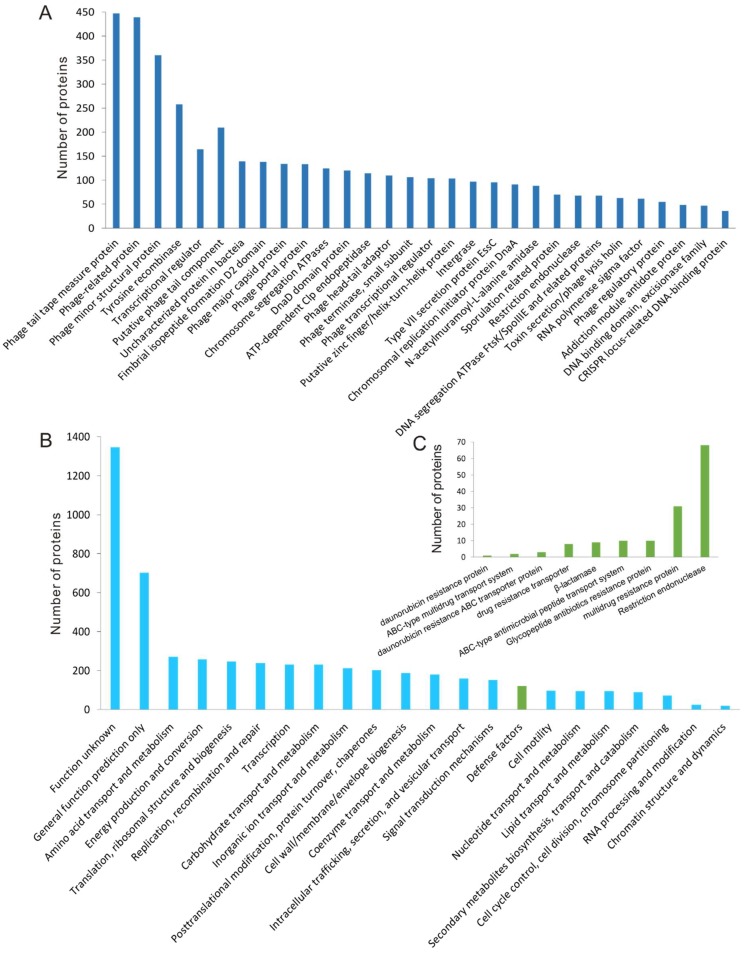
Functional classification of proteins encoded by prophage regions in Bt genomes. (**A**) The proteins related to phage lifecycles were encoded by 135 putative complete prophage genomes. Only proteins with more than 10 homologs of the same function were shown. (**B**) COG classification of proteins encoded by putative complete Bt prophages. (**C**) Functional classification of the proteins that were predicted to take part in the bacterial resistance to antibiotics and exogenous DNA and their corresponding transporter proteins.

The proteins encoded by 135 putative complete prophages were predicted and used for functional classification. Totally, 9477 proteins were predicted, and 5163 of them were homologous with proteins in the COG database. Functional analysis of the prophage proteins revealed that the majority of them were associated with the life cycle of phages, such as structural proteins, DNA replication associated proteins, lytic and lysogenic regulation proteins, and phage host lytic system proteins (Figure 4A,B). The prophage genomes also encoded 97 integrases, which were responsible for the insertion of the temperate phage genomes into the host bacterial genomes, and 70 proteins were annotated as regulators of stationary/sporulation gene expression. 

**Figure 5:**
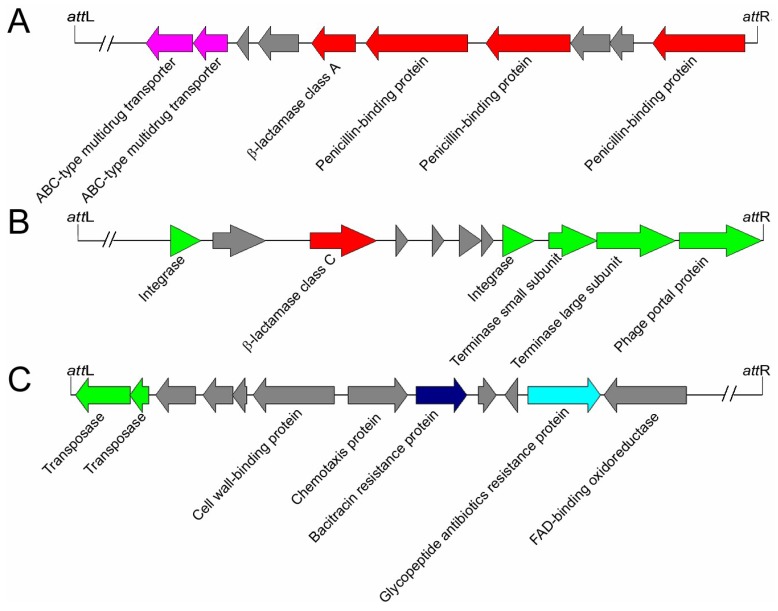
Schematic organization of Bt prophage regions containing antibiotic resistance genes. (**A**) The β-lactamase, ABC-type multidrug transporters, and penicillin-binding proteins encoded by HD-771 prophage-2. (**B**) The β-lactamase encoded by HD-521 prophage-1. (**C**) The bacitracin resistant proteins and glycopeptide antibiotic resistant protein encoded by prophage-12 in the Pasteur Institute Standard strain genome. The proteins associated with the life cycles of phages are indicated in green, and genes not associated with phage life cycles are shown in gray.

In addition, many proteins associated with the phage defense mechanism encoded by bacteria were found in the prophage regions (Figure 4C). Totally, 68 proteins were predicted to be restriction endonucleases. The restriction endonuclease is a member of bacterial restriction-modification (R-M) and plays an important role in giving the bacteria resistance to the exogenous DNA [46]. Additionally, many antibiotics resistance-associated proteins were also found in the prophage genomes. For example, β-lactamase, which is responsible for the resistance of the bacteria to β-lactam antibiotics [47], was found in HD-771 prophage-2, MC-28 prophage-8, HD-521 prophage-1 (Figure 5), L-7601 prophage-3, SCG04-02 prophage-1, Bt185 prophage-3, and BGSC 4AJ1 prophage-8. The alignment of beta-lactamase in HD-771 prophage-2 showed a similarity of 39.2% (79% coverage) with the beta-lactamase (PDB database accession number: 1btl) from *Escherichia coli*. These two beta-lactamases possess identical catalytic site residues (Figure S8). Some proteins, such as glycopeptide antibiotics (10 proteins), daunorubicin (one protein), and their related transporters (13 proteins) were also found in the prophage genomes (Figure 4C).

### 3.4. High Inducibility of Bt Prophages

**Figure 6:**
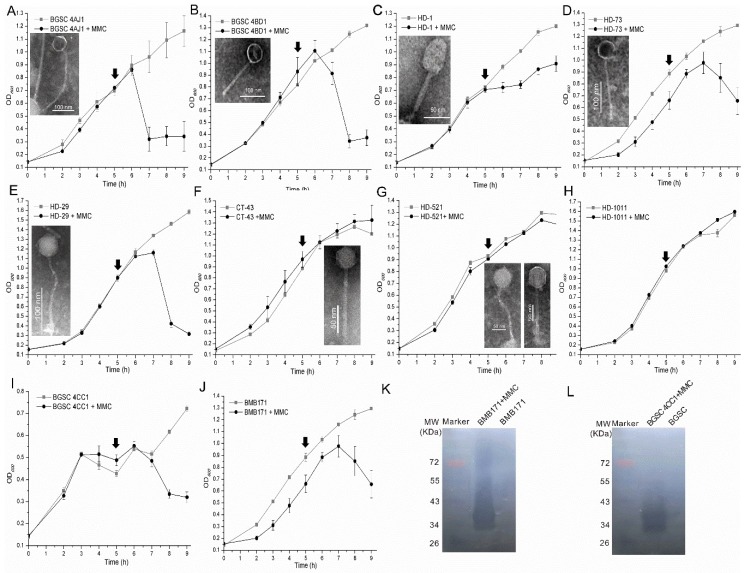
Induction of prophages from Bt strains and characterization of the induced phages. 10 Bt strains, containing putative complete prophage regions, were induced using mitomycin C. Growth curves were plotted (**A**–**J**). The strains without induction with mitomycin C were used as controls and referred to as the names of the strains, while the strains induced by mitomycin C were indicated by adding MMC to the name. The observed phage virions in the supernatants of the strains were shown (**A**–**G**). The lytic activity assays of the culture supernatants of strains (**K**) BMB171 and (**L**) 4CC1 were performed using the in-gel approach using the sterilized host cells. The time points of the addition of mitomycin are indicated by black arrows.

The putative complete prophages contained almost all the genes that were essential for the phage lytic lifecycle and suggesting that they might be induced from bacteria. To analyze the inducibility of the prophages in Bt genomes, 10 Bt strains, including the 4AJ1, 4BD1, HD-1, HD-29, HD-73, HD-521, BMB171, 4CC1, CT-43, and HD-1011 strains, were induced by using mitomycin C, and seven of them exhibited significant changes in the growth. There were no significant changes in growth curves of the HD-521, CT-43, and HD1011 strains (Figure 6). To further analyze the existences of induced phages, the infective activities of the 10 bacterial culture supernatants were detected. The results showed that nine of the ten strain culture supernatants could form lysis zones on the bacterial lawn of Bt strains. Among these nine strains, the culture supernatants of the 4CC1, 4AJ1, HD-1, 4BD1, HD521, and BMB171 strains showed broad lytic ranges and could lyse 31, 41, 21, 18, 33 of the 45 tested Bt strains, separately (Table S5). The incompatibility of phages can inhibit the infection of phages with highly similar genomes due to superinfection exclusion [48,49]. One interesting finding in this study is that the phages (or phage) induced from the 4AJ1 strain could infect the original 4AJ1 strain, which might be because the complete prophage in strain 4AJ1 lacked the mechanism that could provide the strain with resistance to the induced phage. 

The culture supernatants of the 10 strains were detected by using TEM, and phage virions were observed in seven Bt strains (BGSC 4AJ1, BGSC 4BD1, HD-1, HD-29, HD-73, HD-521, and CT-43), including two phages with different morphologies induced from the HD-521 strain. The morphological analysis showed that all the induced phages belonged to the *Siphoviridae* family (Figure 6), which corresponds with previous analysis of Bt inducible prophages [23]. Several phages have extremely long tails, such as the phages induced from the 4AJ1 (approximately 340 nm) and HD-73 (approximately 192 nm) strains. These were longer than many phages infecting Bt [39] and may be novel kinds of phages. Though one putative complete prophage existed in the genome of strain HD-1011, no induced prophage was found using both an infective activity assay and phage virion observation (Figure 6H). The culture supernatants of the other two strains (BMB171 and BGSC 4CC1), which both contained one putative complete prophage in their genomes, showed decreased culture turbidities after the addition of mitomycin C. They also caused lysis zones formation in 10 and 33 of the 45 Bt indicator strains, respectively, but had no observed phage particles or phage-tail-like bacteriocin. Meanwhile, the lysis zones showed no replication ability. Aside from phages, lysin can also lyse the bacteria and generate lysis zones [50,51]. To identify the existence of lysin in the induced culture supernatants of the BMB171 and BGSC 4CC1 strains, in-gel lytic activity assay was used. The results showed that the culture supernatants of the two strains could lyse the host cells (Figure 6K,L), while no lytic activity was observed in the culture supernatants of the two strains that were not induced by mitomycin C. The results indicate that the addition of mitomycin might induce the formation of lysin and further cause the lysis of the indicator strains. 

One induced phage from the HD73 strain was sequenced, and the genome was obtained with 398× fold coverage. About 77,460 high-quality reads were obtained. The average length of the reads was 294 bp. The genome was 42,446 bp in length and consistent with HD73 prophage-1 with 99% identity (99% coverage). The titration of the recovered phage was 10^10^ viron/mL. The phage, which was named phiHD73, was similar with *Bacillus* phage phi4J1, with a similarity of 32.27% in PASC. This phage was classified into the TP21-L-like group using phylogenetic analysis (Figure S5).

## 4. Discussion

The diversity of prophage regions in Bt strains seems higher than in other bacteria, such as the *Pneumococcus* and *Desulfovibrio* genii. Overall, 689 prophage-like fragments were identified from 482 pneumococcal strains, and the average number in each strain was 1.45 [25], and 128 prophage-like elements were screened in 46 of the 47 analyzed genomes (with an average number of 2.72) [26]. Additionally, 398 candidate prophage regions were predicted in 61 Bt strains, and the mean number was approximately 6.52. The temperate phages were reported to contribute to bacterial fitness in at least three ways, including the introduction of fitness factors, gene disruption, and lysis-mediated competitiveness. These are assumed to be the results of the adaptive evolution process of bacteria [52,53]. The antibiotic resistant associated genes in prophage genome regions provide resources for phages to deliver antibiotic resistant genes, and the acquisition of antibiotic resistance genes from phages can increase the environmental tolerance of the bacteria [54]. In *E.coli* K-12, prophage genes were proven to increase host resistance to 11 β-lactam antibiotics, including the most common penicillins and cephalosporins [47]. The bacteria defense systems upon phages include restriction-modification (R-M) systems, CRISPR-Cas, abortive infection systems (Abi), and additional systems such as BREX, prokaryotic Argonautes (pAgos) and DISARM [55]. Restriction endonuclease is a member of the R-M system and protects bacteria from the invasion of phages or the insertion of exogenous DNA [56]. In addition to the benefits mentioned above, prophages can also provide bacteria with resistance to phages that contain similar genome sequences by superinfection exclusion [57,58,59]. Bacteria, like *Bacillus*, that can survive in harsh environments must also be prepared to confront adverse growth conditions. Any resistance factor or metabolic superiority offered by prophages is of great significance and can heighten the rivalrousness of host cells [60].

The contamination of phages during Bt fermentation causes severe losses in yields, and can sometimes cause a failure of fermentation. The findings of this study reveal that putative complete prophages could be induced by mitomycin C. A previous study on Bt temperate phages revealed that the prophages in Bt strains could spontaneously enter the lytic cycle at a high frequency without induction [23]. Combined with the findings of this study, it is rational to speculate that the transformation of prophages from lysogenic form to lytic form might be one of the reasons for Bt phage contamination during fermentation. The excision of prophages can lyse part of the host strains from the inside out. In addition, the lysin generated by prophage can lyse the other strains outside-in, which will aggravate the contamination during fermentation. This may also be the reason the extrinsic approach for controlling phage contamination is of low efficiency. 

The construction of Bt engineering strains is one of the most important ways to improve fermentation properties [61]. Nowadays, several Bt acrystalliferous mutants, such as the BMB171 and HD73 strains, were used as recipient bacteria for constructing Bt engineering strains. Both the BMB171 and HD73 strains, which contained inducible prophages or inducible lysin genes, increased the risk of phage contamination during fermentation. The genome comparison between phiHD73 and prophages in resistant and susceptive Bt strains (Figure S6) showed that transcription regulators and transposases played important roles in superinfection immunity. The comparison of phiHD73 with prophages in resistant Bt strains showed that, except for structural proteins (tail fiber, minor structural protein), the similar prophages in resistant strains all encoded transcriptional regulators. PadR-like family transcription regulators from bacteria mainly participated in the virulence regulation and the efflux mechanism of antibiotics [62]. ArpU family proteins were proven to be late transcriptional regulators in controlling phage-encoded packaging and lysis modules [63]. The genome comparison of phiHD73 with prophages in the susceptive Bt strain, HD-29, which could be infected by the induced phage phiHD73, revealed that the similar prophages in HD-29 stain lacked transposase. Transposase-mediated DNA transposition was reported not only to generate gene diversity but was also reported to be immune to further insertions of the same transposition (transposition immunity) [64,65,66]. Based on the findings of this study, it is possible to design the recipient Bt strains with a genetically modified prophage region that lack lytic regulatory elements and lysin encoding genes but still have transcription regulators and transposases.

## Supplementary Materials

The following are available online at https://www.mdpi.com/1999-4915/11/4/388/s1, Table S1. Strains used for prophage prediction; Table S2. Prophages identified in Bt genomes; Table S3. Putative complete prophages identified in Bt genomes; Table S4. *Bacillus* phages used for pairwise comparison; Table S5. Host range of phages induced from Bt strains; Figure S1. Phylogenetic analysis of Bt prophages using the major capsid proteins; Figure S2. Phylogenetic analysis of Bt prophages using the terminase large subunits; Figure S3. Comparative genomic analysis of prophage from the Bt YBT-1518 strain; Figure S4. Comparative genomic analysis of prophage from the Bt Pasteur Institute Standard strain; Figure S5. Phylogenetic analysis of 41 *Siphoviridae* family phages; Figure S6. The genome comparison between induced phage phiHD73 and candidate prophages in resistant and susceptive Bt strains using Blastn; Figure S7. The amino acid sequences alignment of beta-lactamase in HD-521 prophage-1 with the beta-lactamase (PDB database accession number: 1yqs) from *Streptomyces* R61; Figure S8. The amino acid sequences alignment of beta-lactamase in HD-771 prophage-2 with the beta-lactamase (PDB database accession number: 1btl) from *Escherichia coli*; Figure S9. The amino acid sequences alignment of glycopeptide resistance protein in Pasteur Institute Standard strain prophage-12 with the glycopeptide resistance protein (PDB database accession number: 2dln) from *Escherichia coli*.
