# Supplementary material for: Prevalence and Diversity Analysis of Candidate Prophages to Provide An Understanding on Their Roles in Bacillus Thuringiensis
Source: Viruses. 2019 Apr 25;11(4):388. doi: 10.3390/v11040388 (PMC6521274; doi:10.3390/v11040388)
Supplement: Supplementary file 1 [file viruses-11-00388-s001.pdf]

Prevalence and diversity analysis of candidate prophages to provide an understanding on their roles in *Bacillus thuringiensis*

Yajuan Fu[1,2,+], Yan Wu[1,+], Yihui Yuan[1,3,*] and Meiying Gao[1,*]

[1]Wuhan Institute of Virology, Chinese Academy of Sciences, Wuhan 430071, P. R. China;

[2]University of Chinese Academy of Sciences, Beijing 100039, P. R. China;

[3] Present address: State Key Laboratory of Marine Resource Utilization in South China Sea, Hainan University, Haikou, P. R. China.

[+]These authors contributed equally to this work.

[*]Correspondence and requests for materials should be addressed to Meiying Gao (mygao@wh.iov.cn).

Table 1. Strains used for prophage prediction.

Bt strains	GenBank accession number	Number of prophages			All prophages size(bp)	Ratio in genomes
		Complete	Incomplete	Total		
97-27	NC_005957.1	0	2	2	35201	0.66%
YBT-1518	NC_022873.1	4	10	14	464133	6.96%
Al Hakam	NC_008600.1	0	2	2	30370	0.57%
BMB171	NC_014171.1	1	2	3	83252	1.48%
YBT-020	NC_017200.1	1	3	4	150423	2.65%
CT-43	NC_017208.1	3	5	8	322044	5.24%
HD-771	NC_018500.1	5	5	10	454175	7.05%
HD-789	NC_018508.1	2	3	5	169183	2.67%
MC28	NC_018693.1	3	5	8	255837	3.82%
Bt407-1	NC_018877.1	2	2	4	177905	2.90%
HD73	NC_020238.1	7	1	8	415382	7.03%
IS5056	NC_020376.1	3	5	8	323548	4.78%
YBT-1520	NZ_CP004858.1	4	3	7	360407	5.48%
HD-29	NZ_CP010089.1	4	3	7	304056	4.49%
HD-1	NZ_CP004870.1	2	4	6	303665	4.49%
YBT1520-2	NZ_CP007607.1	3	3	6	341892	5.24%
HD-1011	NZ_CP009335.1	1	3	4	91812	1.51%
HD571	NZ_CP009600.1	0	2	2	23955	0.45%
HD682	NZ_CP009720.1	0	2	2	97612	1.84%
97-27-2	NZ_CP010088.1	0	1	1	14777	0.28%
HD1002	NZ_CP009351.1	2	5	7	283451	4.31%
BGSC 4AA1	NZ_CP010577.1	4	4	8	387450	6.27%
YC-10	NZ_CP011349.1	6	1	7	403515	5.95%
HS18-1	NZ_CP012099.1	1	3	4	127410	1.99%
HD521	NZ_CP010106.1	2	2	4	191673	3.09%
YWC2-8	NZ_CP013055.1	4	4	8	389430	6.25%
CTC	NZ_CP013274.1	0	2	2	22608	0.42%
Pasteur Institute Standard	NZ_AP014864.1	8	7	15	699376	10.18%
Bt185	NZ_CP014282.1	3	0	3	162720	2.55%

HD12	NZ_CP014847.1	5	6	11	485701	7.48%
Bt407-1	NZ_CM000747.1	1	4	5	208507	3.46%
HD1	NZ_CP010005.1	4	3	7	402407	5.87%
BGSC 4Y1	NZ_CM000746.1	3	3	6	202145	3.59%
T01001	NZ_CM000748.1	1	9	10	290537	4.59%
T03a001	NZ_CM000751.1	0	10	10	224034	4.05%
BGSC 4AJ1	NZ_CM000752.1	1	8	9	207065	3.19%
ATCC 10792-1	NZ_CP021061.1	1	6	7	322324	5.15%
BGSC 4AW1	NZ_CM000754.1	0	4	4	154262	2.81%
BGSC 4BA1	NZ_CM000755.1	1	4	5	127755	2.12%
BGSC 4BD1	NZ_CM000756.1	1	4	5	107046	1.72%
BGSC 4CC1	NZ_CM000757.1	1	5	6	125476	2.09%
IBL 200	NZ_CM000758.1	1	4	5	143406	2.13%
IBL 4222	NZ_CM000759.1	1	8	9	236759	3.58%
DAR 81934	NZ_CM001804.1	0	1	1	12836	0.22%
T04001	CM000749.1	1	18	19	452225	7.40%
Bc601	NZ_CP015150.1	5	1	6	346418	5.67%
BGSC 4C1	NZ_CP015176.1	3	4	7	315900	5.43%
MYBT18246	NZ_CP015350.1	5	10	15	678847	10.05%
KNU-07	NZ_CP016588.1	0	3	3	157672	2.56%
Bt18247	NZ_CP015250.1	4	5	9	408728	6.66%
L-7601	NZ_CP020002.1	2	8	10	426869	6.77%
YGd22-03	NZ_CP019230.1	2	3	5	207572	3.29%
SCG04-02	NZ_CP017577.1	1	1	2	34046	0.58%
BM-BT15426	NZ_CP020723.1	0	2	2	60782	1.16%
c25	NZ_CP022345.1	2	2	4	164205	2.90%
ATCC 10792-2	NZ_CM000753.1	1	9	10	278757	4.45%
ST7	CP016194.1	3	5	8	302253	4.81%
ATCC 10792-3	CP020754.1	2	5	7	331464	5.14%
XL6	CP013000.1	1	3	4	164549	2.89%
T13001	NZ_CM000750.1	1	9	10	301419	4.99%
LM1212	CP024771.1	6	2	8	349306	5.53%

Table 2. Candidate prophages regions identified in Bt genomes.

Prophages	Total length (bp)	Positions in bacteria chromosome		Type of prophage	Best match phages	Coverage (%)	Identity (%)
		Start	End				
97-27 prophage-1	14940	1009871	1024810	incomplete			
97-27 prophage-2	20261	1199512	1219772	incomplete			
YBT-1518 prophage-1	42889	257730	300618	incomplete	*Bacillus* phage vB_BtS_BMBtp15	42	93.32
YBT-1518 prophage-2	31054	415206	446259	incomplete	*Bacillus* phage vB_BtS_BMBtp16	100	99.79
YBT-1518 prophage-3	31039	772058	803096	incomplete	*Bacillus* phage BMBtp1	8	90.23
YBT-1518 prophage-4	18763	848635	867396	incomplete	*Bacillus* phage vB_BtS_BMBtp16	78	90.56
YBT-1518 prophage-5	33609	882892	916500	complete	*Bacillus* phage BtCS33	67	94.13
YBT-1518 prophage-6	20482	1443308	1463789	incomplete			
YBT-1518 prophage-7	57001	1788360	1845360	incomplete	*Bacillus* phage Carmel_SA	17	91.8
YBT-1518 prophage-8	31727	2572024	2603750	incomplete	*Bacillus* phage phiCM3	5	83.53
YBT-1518 prophage-9	6980	3044091	3051070	incomplete			
YBT-1518 prophage-10	52143	4260401	4312543	complete	*Bacillus* phage 11143	15	85.62
YBT-1518 prophage-11	23854	4623183	4647036	incomplete	*Bacillus* phage BMBtp1	13	83.58
YBT-1518 prophage-12	40309	5316310	5356618	complete	*Bacillus* phage phi4I1	44	88.08
YBT-1518 prophage-13	40120	5517648	5557767	complete	*Bacillus* phage phi4I1	42	85.93
YBT-1518 prophage-14	34163	5647562	5681724	incomplete	*Bacillus* phage vB_BtS_BMBtp15	52	92.79
Al Hakam prophage-1	20381	1215373	1235753	incomplete			

Al Hakam prophage-2	9989	2364594	2374582	incomplete			
BMB171 prophage-1	20479	1196392	1216870	incomplete			
BMB171 prophage-2	45513	2498467	2543969	complete	*Bacillus* phage phiCM3	15	90.09
BMB171 prophage-3	17260	3580569	3597828	incomplete	*Bacillus* phage vB_BtS_BMBtp3	4	77.57
YBT-020 prophage-1	53160	390634	443893	complete	*Bacillus* phage vB_BceS-MY192	16	85.46
YBT-020 prophage-2	42537	1115027	1157563	incomplete	*Bacillus* phage phi4B1	90	100
YBT-020 prophage-3	15145	1352176	1367320	incomplete			
YBT-020 prophage-4	39581	4721857	4761437	incomplete	*Bacillus* phage vB_BtS_BMBtp16	37	91.88
CT-43 prophage-1	39114	694975	734088	incomplete	*Bacillus* phage phiCM3	14	98.09
CT-43 prophage-2	13004	1021860	1034863	incomplete			
CT-43 prophage-3	14886	1224209	1239094	incomplete			
CT-43 prophage-4	84201	1826113	1910313	complete	*Bacillus* phage BceA1 /*Staphylococcus* phage SpaA1	7	89.15
CT-43 prophage-5	19645	2350991	2370635	incomplete	*Bacillus* phage BtCS33 /*Bacillus* phage phi4I1 /*Bacillus* phage BtiUFT6.51-F	9	83.3
CT-43 prophage-6	66379	2588715	2655093	complete	*Bacillus* phage BVE2	5	89.27
CT-43 prophage-7	43658	3773368	3817025	complete	Uncultured *Caudovirales* phage clone 3F_8	24	84.4
CT-43 prophage-8	41157	4904766	4945922	incomplete	*Bacillus* phage vB_BtS_BMBtp16	39	92.73
HD-771 prophage-1	37673	1087752	1125424	imcomplet e	*Bacillus* phage phiS58/*Bacillus* phage phi4J1/*Bacillus* phage Waukesha92/*Bacillus* phage BceA1/*Staphylococcus* phage SpaA1	1	72.41
HD-771 prophage-2	66113	1359225	1425337	complete	*Bacillus* phage vB_BtS_BMBtp13	24	94.34
HD-771 prophage-3	64662	2015855	2080516	imcomplet e	*Bacillus* phage vB_BtS_BMBtp3	10	92.36
HD-771 prophage-4	20301	2852622	2872922	incomplete			
HD-771 prophage-5	60395	2920758	2981152	complete	Uncultured *Caudovirales* phage clone 3F_8	15	84.39
HD-771 prophage-6	39777	4500609	4540385	complete	*Bacillus* phage phi4J1	30	87.76
HD-771 prophage-7	34536	4676595	4711130	incomplete	*Bacillus* phage BMBtp1	73	94.14
HD-771 prophage-8	40354	5195637	5236990	incomplete	*Bacillus* phage vB_BtS_BMBtp15	45	94.81
HD-771 prophage-9	48453	5442197	5490649	complete	Uncultured *Caudovirales* phage clone 7S_1	21	85.51
HD-771 prophage-10	41911	5764272	5806182	complete	*Bacillus* phage BtCS33	30	93.21
HD-789 prophage-1	14805	762327	777131	incomplete			
HD-789 prophage-2	61836	2938754	3000589	complete	*Bacillus* phage phIS3501	71	99.98
HD-789 prophage-3	40530	3050147	3090676	incomplete	*Bacillus* phage phIS3501	9	94.41
HD-789 prophage-4	37849	3328682	3366530	complete	Uncultured *Caudovirales* phage clone 3F_8	29	84.34
HD-789 prophage-5	14163	5293480	5307642	incomplete			
MC28 prophage-1	15006	413630	428635	incomplete			
MC28 prophage-2	17175	1441237	1458411	incomplete	*Bacillus* phage vB_BceS-MY192	6	80.9
MC28 prophage-3	20809	2819914	2840722	incomplete	*Bacillus* phage vB_BtS_BMBtp14	5	81.81
MC28 prophage-4	52670	2934767	2987436	complete	Uncultured *Caudovirales* phage clone 3F_8	24	87.4
MC28 prophage-5	20408	2999188	3019595	incomplete			
MC28 prophage-6	17679	3038714	3056392	incomplete			
MC28 prophage-7	49920	3102093	3152012	complete	*Bacillus* phage PfEFR-5/*Bacillus* phage PfEFR-4	31	87.37
MC28 prophage-8	62170	5095369	5157538	complete	*Bacillus* phage vB_BtS_BMBtp16	10	93.31
Bt407-1 prophage-1	23241	696825	720065	incomplete	*Bacillus* phage phiCM3	8	98.26
Bt407-1 prophage-2	81548	1830538	1912085	complete	*Bacillus* phage BceA1 /*Staphylococcus* phage SpaA1	7	89.15
Bt407-1 prophage-3	26933	2348009	2374941	incomplete	*Bacillus* phage phiCM3	6	83.59
Bt407-1 prophage-4	46183	2605770	2651952	complete	*Bacillus* phage BVE2	8	89.27

HD73 prophage-1	42253	231160	273413	complete	*Bacillus* phage phi4J1	20	94.71
HD73 prophage-2	61572	376296	437867	complete	*Bacillus* phage BceA1 /*Staphylococcus* phage SpaA1	9	99.76
HD73 prophage-3	57693	760542	818234	complete	*Bacillus* phage BtCS33	72	99.97
HD73 prophage-4	55077	1282708	1337784	complete	*Bacillus* phage phi4J1	19	89.02
HD73 prophage-5	20416	1383091	1403506	incomplete			
HD73 prophage-6	54689	2138013	2192701	complete	*Bacillus* phage BtCS33	58	98.84
HD73 prophage-7	58499	5020287	5078785	complete	*Bacillus* phage phIS3501	63	95.02
HD73 prophage-8	44192	5232007	5276198	complete	*Bacillus* phage phi4J1	23	92.42
IS5056 prophage-1	39112	692092	731203	incomplete	*Bacillus* phage phiCM3	14	98.09
IS5056 prophage-2	13004	1018979	1031982	incomplete			
IS5056 prophage-3	20264	1216033	1236296	incomplete			
IS5056 prophage-4	79357	1841121	1920478	complete	*Bacillus* phage BceA1 /*Staphylococcus* phage SpaA1	8	89.15
IS5056 prophage-5	20420	2356189	2376608	incomplete	*Bacillus* phage BtCS33/*Bacillus* phage phi4I1/*Bacillus* phage BtiUFT6.51-F	9	83.3
IS5056 prophage-6	66381	2594876	2661256	complete	*Bacillus* phage BVE2	5	89.27
IS5056 prophage-7	43853	3776967	3820819	complete	Uncultured *Caudovirales* phage clone 3F_8	24	84.42
IS5056 prophage-8	41157	4909665	4950821	incomplete	*Bacillus* phage vB_BtS_BMBtp16	39	92.73
YBT-1520 prophage-1	90139	243519	333657	complete	*Bacillus* phage BMBtp1	9	87.57
YBT-1520 prophage-2	58551	756854	815404	complete	*Bacillus* phage phi4I1	59	99.99
YBT-1520 prophage-3	42947	1280945	1323891	complete	*Bacillus* phage phIS3501	4	82.53
YBT-1520 prophage-4	20460	1380714	1401173	incomplete			
YBT-1520 prophage-5	30424	2124300	2154723	incomplete	*Bacillus* phage phIS3501	40	89.22
YBT-1520 prophage-6	78741	4963649	5042389	complete	*Bacillus* phage phIS3501	46	94.97
YBT-1520 prophage-7	39145	5192924	5232068	incomplete	*Bacillus* phage vB_BtS_BMBtp15	46	96.3
HD-29 prophage-1	51911	156843	208753	complete	*Bacillus* phage phi4J1	26	99.59
HD-29 prophage-2	54586	843002	897587	complete	*Bacillus* phage BceA1 /*Staphylococcus* phage SpaA1	75	100
HD-29 prophage-3	32765	2493852	2526617	incomplete	*Bacillus* phage vB_BtS_BMBtp3	14	82.98
HD-29 prophage-4	4296	2992879	2997175	incomplete	*Bacillus* phage vB_BtS_BMBtp14	27	74.93
HD-29 prophage-5	70288	4946878	5017166	complete	*Bacillus* phage BMBtp1	20	93.29
HD-29 prophage-6	46019	5030224	5076243	incomplete	Phage Wrath	31	92.42
HD-29 prophage-7	44191	5200050	5244240	complete	*Bacillus* phage phi4J1	23	92.42
HD-1 prophage-1	66552	258214	324765	complete	*Bacillus* phage phi4J1	15	94.69
HD-1 prophage-2	48464	400116	448579	complete	*Bacillus* phage BceA1 /*Staphylococcus* phage SpaA1	5	99.7
HD-1 prophage-3	40506	781975	822480	incomplete	*Bacillus* phage BceA1 /*Staphylococcus* phage SpaA1	43	96.78
HD-1 prophage-4	53101	1296841	1349941	imcomplete	Uncultured *Caudovirales* phage clone 9AX_2	20	87.3
HD-1 prophage-5	75871	4992087	5067957	imcomplete	*Bacillus* phage phiCM3	39	97.33
HD-1 prophage-6	19171	5225976	5245146	incomplete	*Bacillus* phage phi4J1	22	91.41
YBT1520-2 prophage-1	73308	19909	93216	complete	*Bacillus* phage phi4J1	19	95.86
YBT1520-2 prophage-2	35925	707688	743612	incomplete	*Bacillus* phage BMBtp1	38	93.33
YBT1520-2 prophage-3	78739	894807	973545	complete	*Bacillus* phage phIS3501	46	94.97
YBT1520-2 prophage-4	53561	3791245	3844805	imcomplete	*Bacillus* phage BtCS33	59	98.73
YBT1520-2 prophage-5	38379	4650515	4688893	imcomplete	Uncultured *Caudovirales* phage clone 9AX_2	19	85.81
YBT1520-2 prophage-6	61980	5153713	5215692	complete	*Bacillus* phage BtiUFT6.51-F	67	99.9
HD-1011 prophage-1	14779	2355738	2370516	incomplete			
HD-1011 prophage-2	51810	2910951	2962760	complete	*Bacillus* phage phi4I1	36	89.16

HD-1011 prophage-3	6681	3521183	3527863	incomplete			
HD-1011 prophage-4	18542	4542029	4560570	incomplete			
HD571 prophage-1	9989	103622	113610	incomplete			
HD571 prophage-2	13966	117579	131544	incomplete	*Bacillus* phage vB_BceS-MY192	14	82.64
HD682 prophage-1	67350	1214601	1281950	incomplete	Uncultured *Caudovirales* phage clone 3F_8	7	80.89
HD682 prophage-2	30262	2576180	2606441	incomplete			
97-27-2 prophage-1	14777	1213524	1228300	incomplete			
HD1002 prophage-1	52608	47815	100422	complete	Uncultured *Caudovirales* phage clone 3F_8	21	84.34
HD1002 prophage-2	37194	179210	216403	incomplete			
HD1002 prophage-3	56570	320439	377008	incomplete	*Bacillus* phage phIS3501	7	94.41
HD1002 prophage-4	66757	417583	484339	complete	*Bacillus* phage phIS3501	66	99.98
HD1002 prophage-5	12889	2639455	2652343	incomplete			
HD1002 prophage-6	33240	3597048	3630287	incomplete	Moraxella phage Mcat16	2	79.87
HD1002 prophage-7	24193	5047691	5071883	incomplete	*Bacillus* phage vB_BtS_BMBtp15	32	81.82
BGSC 4AA1 prophage-1	39416	642522	681937	incomplete	*Bacillus* phage BMBtp1	87	100
BGSC 4AA1 prophage-2	57403	1165897	1223299	complete	*Bacillus* phage vB_BtS_BMBtp3	13	99.98
BGSC 4AA1 prophage-3	12835	1269806	1282640	incomplete			
BGSC 4AA1 prophage-4	39650	1917165	1956814	imcomplete	*Bacillus* phage proCM3	94	99.97
BGSC 4AA1 prophage-5	64804	2610167	2674970	incomplete	Uncultured *Caudovirales* phage clone 3F_8	14	84.48
BGSC 4AA1 prophage-6	54220	3902354	3956573	complete	Uncultured *Caudovirales* phage clone 3F_8	16	82.98
BGSC 4AA1 prophage-7	46046	4593770	4639815	complete	*Bacillus* phage Carmel_SA	65	92.36
BGSC 4AA1 prophage-8	73076	5189813	5262888	complete	*Bacillus* phage phi4I1	48	95.78
YC-10 prophage-1	55152	579100	634251	complete	*Bacillus* phage BtCS33	58	98.81
YC-10 prophage-2	39965	1443466	1483430	imcomplete	*Bacillus* phage BMBtp1	22	88.51
YC-10 prophage-3	61975	1949726	2011700	complete	*Bacillus* phage phi4I1	67	100
YC-10 prophage-4	51804	2331105	2382908	complete	*Bacillus* phage vB_BtS_BMBtp16	12	85.99
YC-10 prophage-5	75947	2466727	2542673	complete	*Bacillus* phage phi4J1	16	94.71
YC-10 prophage-6	42540	3157229	3199768	complete	*Bacillus* phage phi4J1	23	92.42
YC-10 prophage-7	76132	3353688	3429819	complete	*Bacillus* phage phiCM3	40	96.57
HS18-1 prophage-1	12812	1194101	1206912	incomplete			
HS18-1 prophage-2	43215	1376234	1419448	incomplete	Uncultured *Caudovirales* phage clone 3F_8	22	87.31
HS18-1 prophage-3	20676	2489077	2509752	incomplete	*Bacillus* phage phiCM3	7	83.97
HS18-1 prophage-4	50707	2496265	2546971	complete	*Bacillus* phage phiCM3	38	87.88
HD521 prophage-1	39615	1988162	2027776	complete	*Bacillus* phage BtCS33	4	80.74
HD521 prophage-2	18844	2028783	2047626	incomplete	*Bacillus* phage vB_BtS_BMBtp15	4	75.35
HD521 prophage-3	88371	2287686	2376056	complete	*Bacillus* phage phiCM3	5	83.54
HD521 prophage-4	44843	3817538	3862380	incomplete	*Bacillus* phage PfEFR-5/*Bacillus* phage PfEFR-4	9	82.83
YWC2-8 prophage-1	48053	1523	49575	imcomplete	*Bacillus* phage phi4J1	8	94.71
YWC2-8 prophage-2	42541	666907	709447	imcomplete	*Bacillus* phage phi4J1	23	92.42
YWC2-8 prophage-3	76135	863370	939504	complete	*Bacillus* phage phiCM3	40	96.58
YWC2-8 prophage-4	55155	3763087	3818241	complete	*Bacillus* phage BtCS33	58	98.81
YWC2-8 prophage-5	39965	4627496	4667460	imcomplete	*Bacillus* phage phi4J1	27	89.47

YWC2-8 prophage-6	61976	5133773	5195748	complete	*Bacillus* phage BtiUFT6.51-F /*Bacillus* phage phiCM3	67	99.98
YWC2-8 prophage-7	51805	5515164	5566968	complete	*Bacillus* phage BceA1 /*Staphylococcus* phage SpaA1	11	99.76
YWC2-8 prophage-8	13800	5656460	5670259	incomplete	*Bacillus* phage phi4J1	55	87.97
CTC prophage-1	14730	1194064	1208793	incomplete			
CTC prophage-2	7878	2512884	2520761	incomplete	*Bacillus* phage BVE2	43	89.83
Pasteur Institute Standard prophage-1	51244	317809	369052	complete	Uncultured *Caudovirales* phage clone 3F_9	20	87.15
Pasteur Institute Standard prophage-2	44877	573162	618038	complete	*Bacillus* phage phIS3501	22	93.34
Pasteur Institute Standard prophage-3	38308	1061766	1100073	incomplete	*Bacillus* phage vB_BtS_BMBtp15	48	96.32
Pasteur Institute Standard prophage-4	98877	1295431	1394307	complete	*Bacillus* phage PfEFR-5	22	90.11
Pasteur Institute Standard prophage-5	68615	1913253	1981867	complete	*Bacillus* phage phiCM3	8	77.79
Pasteur Institute Standard prophage-6	51422	2086890	2138311	complete	*Bacillus* phage BtCS33	59	96.21
Pasteur Institute Standard prophage-7	7903	3247335	3255237	incomplete			
Pasteur Institute Standard prophage-8	40112	3799140	3839251	imcomplete	*Bacillus* phage proCM3	89	95.71
Pasteur Institute Standard prophage-9	67089	4006836	4073924	imcomplete			
Pasteur Institute Standard prophage-10	34743	4608461	4643203	incomplete	*Bacillus* phage BMBtp1	51	94.79
Pasteur Institute Standard prophage-11	42578	4661441	4704018	complete	*Bacillus* phage phi4J1	24	88.95
Pasteur Institute Standard prophage-12	61607	5100297	5161903	complete	*Bacillus* phage phi4I1	68	99.95
Pasteur Institute Standard prophage-13	9769	5357908	5367676	incomplete			
Pasteur Institute Standard prophage-14	50429	5512767	5563195	complete	*Bacillus* phage BtCS33	22	94.72
Pasteur Institute Standard prophage-15	31803	5624567	5656369	imcomplete	Dickeya phage phiDP10.3 clone pD10	11	76.35
Bt185 prophage-1	65736	1760889	1826624	complete	*Bacillus* phage phiCM3	8	84.08
Bt185 prophage-2	62376	2479645	2542020	complete	*Bacillus* phage vB_BtS_BMBtp13	30	91.68
Bt185 prophage-3	34608	4251904	4286511	complete	Uncultured *Caudovirales* phage clone 3F_9	60	88.61
HD12 prophage-1	68685	328204	396888	complete	Uncultured *Caudovirales* phage clone 7S_1	16	86.23
HD12 prophage-2	38777	1197527	1236303	incomplete	*Bacillus* phage phi4J1	31	89.48
HD12 prophage-3	12835	1291598	1304432	incomplete			
HD12 prophage-4	43771	1936887	1980657	imcomplete	*Bacillus* phage proCM3	83	95.71
HD12 prophage-5	56821	2084122	2140942	complete	*Bacillus* phage phIS3501	58	94.41
HD12 prophage-6	30764	2713097	2743860	incomplete	Uncultured *Caudovirales* phage clone 3F_8	20	84.88
HD12 prophage-7	17322	2912967	2930288	incomplete			
HD12 prophage-8	38935	3865425	3904359	incomplete	Uncultured *Caudovirales* phage clone 9AX_2	1	82.71

HD12 prophage-9	43010	5097838	5140847	complete	*Bacillus* phage BMBtp1	38	90.41
HD12 prophage-10	77355	5284393	5361747	complete	*Bacillus* phage phiCM3	50	99.76
HD12 prophage-11	57426	5399779	5457204	complete	*Bacillus* phage vB_BtS_BMBtp15	46	95.39
Bt407-1 prophage-1	39111	623829	662940	incomplete	*Bacillus* phage phiCM3	14	98.09
Bt407-1 prophage-2	9016	941846	950862	incomplete			
Bt407-1 prophage-3	20444	1134828	1155272	incomplete			
Bt407-1 prophage-4	10702 9	3627284	3734313	complete	Uncultured *Caudovirales* phage clone 3F_8	10	84.4
Bt407-1 prophage-5	32907	4619642	4652549	incomplete	*Bacillus* phage vB_BtS_BMBtp16	49	92.73
HD1 prophage-1	42540	550875	593415	incomplete	*Bacillus* phage phi4J1	23	92.42
HD1 prophage-2	76133	747338	823471	complete	*Bacillus* phage phIS3501	48	95.02
HD1 prophage-3	55153	3647841	3702994	complete	*Bacillus* phage BtCS33	58	98.82
HD1 prophage-4	39964	4512247	4552211	incomplete	*Bacillus* phage phi4J1	27	89.47
HD1 prophage-5	52167	5028288	5080455	complete	*Bacillus* phage phi4I1	80	99.99
HD1 prophage-6	60502	5400392	5460894	incomplete	*Bacillus* phage BceA1 /*Staphylococcus* phage SpaA1	10	99.76
HD1 prophage-7	75948	5535541	5611489	complete			
BGSC 4Y1 prophage-1	21536	1078124	1099660	incomplete			
BGSC 4Y1 prophage-2	25458	3365884	3391342	incomplete	Phage Wrath	3	91.19
BGSC 4Y1 prophage-3	52658	3400388	3453046	complete	Uncultured *Caudovirales* phage clone 3F_8	9	83.93
BGSC 4Y1 prophage-4	35726	3720053	3755779	complete	*Bacillus* phage BtiUFT6.51-F	42	88.55
BGSC 4Y1 prophage-5	18952	3885353	3904305	incomplete	*Bacillus* phage BVE2	32	90.24
BGSC 4Y1 prophage-6	47815	4052653	4100468	complete	*Bacillus* phage PfEFR-5	44	99.31
T01001 prophage-1	39112	619339	658450	incomplete	*Bacillus* phage phiCM3	14	98.09
T01001 prophage-2	15104	1099256	1114359	incomplete			
T01001 prophage-3	70184	3621373	3691545	incomplete	*Bacillus* phage BceA1 /*Staphylococcus* phage SpaA1	9	89.15
T01001 prophage-4	11644	5382584	5394227	incomplete	*Bacillus* phage BMBtp1	3	81.35
T01001 prophage-5	35750	5397285	5433020	incomplete	*Bacillus* phage vB_BtS_BMBtp13	5	89.35
T01001 prophage-6	9114	5745011	5754124	incomplete			
T01001 prophage-7	9017	5840688	5849704	incomplete			
T01001 prophage-8	35640	6025724	6061363	incomplete	*Bacillus* phage vB_BtS_BMBtp16	47	92.73
T01001 prophage-9	38182	6161127	6199297	complete	Uncultured *Caudovirales* phage clone 3F_8	28	84.4
T01001 prophage-10	26790	6241373	6268151	incomplete	*Bacillus* phage phiNIT1	0	90.1
T03a001 prophage-1	22524	205376	227900	incomplete	*Bacillus* phage phi4J1	16	94.71
T03a001 prophage-2	6625	647481	654106	incomplete	*Bacillus* phage BtCS33	100	100
T03a001 prophage-3	20414	1082478	1102892	incomplete			
T03a001 prophage-4	16518	5002022	5018540	incomplete	Uncultured *Caudovirales* phage clone 7S_1	51	84.41
T03a001 prophage-5	4468	5021281	5025749	incomplete	*Bacillus* phage phIS3501	39	95.23
T03a001 prophage-6	14829	5065591	5080420	incomplete	Uncultured *Caudovirales* phage clone 3F_8	34	82.35
T03a001 prophage-7	11221	5087153	5098374	incomplete	*Bacillus* phage phIS3501	78	92.87
T03a001 prophage-8	17786	5219845	5237631	incomplete	*Bacillus* phage phIS3501	32	96.47
T03a001 prophage-9	36889	5362759	5399648	incomplete	*Bacillus* phage phIS3501	15	94.99
T03a001 prophage-10	72760	5441613	5514373	incomplete	*Bacillus* phage phiCM3	18	95.13
BGSC 4AJ1 prophage-1	16285	1096472	1112757	incomplete			
BGSC 4AJ1 prophage-2	25952	3379639	3405591	incomplete	*Bacillus* phage vB_BtS_BMBtp3	43	94.46
BGSC 4AJ1 prophage-3	15313	4303255	4318568	incomplete	*Bacillus* phage vB_BtS_BMBtp15	1	91.33
BGSC 4AJ1 prophage-4	24966	5370638	5395604	incomplete	*Bacillus* phage 250	1	82.45
BGSC 4AJ1 prophage-5	12048	5677945	5689993	incomplete	*Bacillus* phage PfIS075/*Bacillus* phage PfNC7401	35	92.34
BGSC 4AJ1 prophage-6	16864	6197528	6214392	incomplete	*Bacillus* phage vB_BtS_BMBtp14	100	100
BGSC 4AJ1 prophage-7	29105	6228308	6257413	incomplete	*Bacillus* phage vB_BtS_BMBtp14	39	100
BGSC 4AJ1 prophage-8	47868	6380454	6428322	complete	*Bacillus* phage phIS3501	37	89.53

BGSC 4AJ1 prophage-9	18664	6447146	6465810	incomplete	*Bacillus* phage PfIS075/*Bacillus* phage PfNC7401	9	90.04
ATCC 10792-1 prophage-1	45060	529088	574148	incomplete	*Bacillus* phage phiCM3	14	98.09
ATCC 10792-1 prophage-2	12840	1070068	1082908	incomplete			
ATCC 10792-1 prophage-3	76649	1672195	1748844	incomplete	*Bacillus* phage BceA1 /*Staphylococcus* phage SpaA1	8	89.15
ATCC 10792-1 prophage-4	29367	2189829	2219196	incomplete	*Bacillus* phage BtiUFT6.51-F/*Bacillus* phage phi4I1/*Bacillus* phage BtCS33	6	83.3
ATCC 10792-1 prophage-5	66379	2436315	2502694	complete	*Bacillus* phage BVE2	5	89.27
ATCC 10792-1 prophage-6	50430	3626948	3677378	incomplete	Uncultured *Caudovirales* phage clone 3F_8	21	84.42
ATCC 10792-1 prophage-7	41599	4760216	4801815	incomplete	*Bacillus* phage vB_BtS_BMBtp16	39	92.73
BGSC 4AW1 prophage-1	12408	1072703	1085111	incomplete			
BGSC 4AW1 prophage-2	29179	1744495	1773674	incomplete	*Bacillus* phage phIS3501	32	86.59
BGSC 4AW1 prophage-3	58109	5191126	5249235	incomplete	*Bacillus* phage phIS3501	12	92.13
BGSC 4AW1 prophage-4	54566	5297892	5352458	incomplete	*Bacillus* phage PfNC7401	11	87.92
BGSC 4BA1 prophage-1	16475	1080794	1097269	incomplete			
BGSC 4BA1 prophage-2	46913	1603487	1650400	complete	*Bacillus* phage phIS3501	44	89.41
BGSC 4BA1 prophage-3	6629	2204935	2211564	incomplete			
BGSC 4BA1 prophage-4	11085	3180277	3191362	incomplete	*Bacillus* phage vB_BtS_BMBtp14	8	81.93
BGSC 4BA1 prophage-5	46653	5143458	5190111	incomplete	*Bacillus* phage BVE2	3	88
BGSC 4BD1 prophage-1	14795	1080344	1095139	incomplete			
BGSC 4BD1 prophage-2	31750	1897694	1929444	incomplete	*Bacillus* phage phiCM3	6	83.2
BGSC 4BD1 prophage-3	31210	3543477	3574687	complete	Uncultured *Caudovirales* phage clone 3F_8	37	84.84
BGSC 4BD1 prophage-4	11304	5709665	5720969	incomplete			
BGSC 4BD1 prophage-5	17987	5760274	5778261	incomplete	*Bacillus* phage phiS58	5	84.93
BGSC 4CC1 prophage-1	20210	1120594	1140804	incomplete			
BGSC 4CC1 prophage-2	50410	2381307	2431717	complete	Uncultured *Caudovirales* phage clone 9AX_2	38	88.85
BGSC 4CC1 prophage-3	18987	3401242	3420229	incomplete	*Bacillus* phage vB_BtS_BMBtp14	5	82.05
BGSC 4CC1 prophage-4	12735	5444130	5456865	incomplete	*Bacillus* phage PfEFR-5/*Bacillus* phage PfEFR-4	3	75.82
BGSC 4CC1 prophage-5	12159	5604687	5616846	incomplete			
BGSC 4CC1 prophage-6	10975	5840515	5851490	incomplete			
IBL 200 prophage-1	14882	1108155	1123037	incomplete			

IBL 200 prophage-2	16981	4461372	4478353	incomplete			
IBL 200 prophage-3	22624	5560789	5583413	incomplete	*Bacillus* phage phiCM3	4	86.81
IBL 200 prophage-4	50472	6106610	6157082	complete	*Bacillus* phage BMBtp1	23	92.14
IBL 200 prophage-5	38447	6169806	6208253	incomplete	*Bacillus* phage vB_BtS_BMBtp16	37	97.07
IBL 4222 prophage-1	30230	366046	396276	incomplete	*Bacillus* phage phIS3501	52	99.99
IBL 4222 prophage-2	36557	3222822	3259379	incomplete	*Bacillus* phage phiCM3	3	89.42
IBL 4222 prophage-3	28706	5441836	5470542	incomplete			
IBL 4222 prophage-4	20470	5467483	5487953	incomplete	*Bacillus* phage phIS3501	83	99.99
IBL 4222 prophage-5	12794	5516015	5528809	incomplete	*Bacillus* phage phIS3501	58	100
IBL 4222 prophage-6	14227	5617122	5631349	incomplete	*Bacillus* phage TsarBomba	5	79.02
IBL 4222 prophage-7	33486	5648777	5682263	incomplete			
IBL 4222 prophage-8	45827	5981134	6026961	complete	Uncultured *Caudovirales* phage clone 3F_8	24	84.34
IBL 4222 prophage-9	14462	6552047	6566509	incomplete			
DAR 81934 prophage-1	12836	1394932	1407768	incomplete			
T04001 prophage-1	26878	275340	302218	incomplete	*Bacillus* phage vB_BtS_BMBtp16	7	92.97
T04001 prophage-2	36835	389552	426387	incomplete	*Bacillus* phage BtiUFT6.51-F /*Bacillus* phage phi4I1/*Bacillus* phage BtCS33/*Bacillus* phage BceA1/*Staphylococcus* phage SpaA1	7	76.81
T04001 prophage-3	12902	1069979	1082881	incomplete			
T04001 prophage-4	25493	3156199	3181692	incomplete	Uncultured *Caudovirales* phage clone 3F_8	27	84.86
T04001 prophage-5	32770	3422790	3455560	complete	*Bacillus* phage BtCS33	31	94.4
T04001 prophage-6	12934	3459224	3472158	incomplete	*Bacillus* phage PfEFR-5/*Bacillus* phage PfEFR-4	4	85.11
T04001 prophage-7	23158	3810649	3833807	incomplete	*Bacillus* phage PfEFR-5	10	88.28
T04001 prophage-8	18233	3976107	3994340	incomplete	Uncultured *Caudovirales* phage clone 3F_9	18	82.57
T04001 prophage-9	25537	4550999	4576536	incomplete	Streptococcus phage 20617	5	74.83
T04001 prophage-10	27308	4622014	4649322	incomplete	Uncultured *Caudovirales* phage clone 7S_4	18	83.54
T04001 prophage-11	25439	4999236	5024675	incomplete	*Bacillus* phage BMBtp1	2	85.74
T04001 prophage-12	40589	5045354	5085943	incomplete	*Bacillus* phage BMBtp1	3	92.01
T04001 prophage-13	36097	5219160	5255257	incomplete	*Bacillus* phage BMBtp1	22	97.67
T04001 prophage-14	7499	5264088	5271587	incomplete			
T04001 prophage-15	15902	5305648	5321550	incomplete	*Bacillus* phage IEBH	29	86.93
T04001 prophage-16	15796	5369312	5385108	incomplete	Uncultured *Caudovirales* phage clone 7S_1	17	82.7
T04001 prophage-17	17326	5549515	5566841	incomplete	*Bacillus* phage Waukesha92	6	81.42
T04001 prophage-18	34982	5718999	5753981	incomplete	Uncultured *Caudovirales* phage clone 7S_1	31	85.5
T04001 prophage-19	16547	6061501	6078048	incomplete	*Bacillus* phage vB_BtS_BMBtp3	7	93
Bc601 prophage-1	75948	248980	324928	complete	*Bacillus* phage phi4J1	16	94.71
Bc601 prophage-2	54134	730098	784232	complete	*Bacillus* phage phi4I1	77	100
Bc601 prophage-3	39964	1255558	1295522	incomplete	*Bacillus* phage phi4J1	27	89.47
Bc601 prophage-4	56178	2107525	2163703	complete	*Bacillus* phage BtCS33	59	98.89
Bc601 prophage-5	74433	4985834	5060267	complete	*Bacillus* phage phIS3501	47	95.03
Bc601 prophage-6	45761	5210826	5256587	complete	*Bacillus* phage phi4J1	21	92.42
BGSC 4C1 prophage-1	46652	649148	695800	complete	*Bacillus* phage phi4J1	29	90.14
BGSC 4C1 prophage-2	45810	1020073	1065883	complete	*Bacillus* phage BMBtp1	26	90.8
BGSC 4C1 prophage-3	12824	1236282	1249106	incomplete			
BGSC 4C1 prophage-4	64576	2304587	2369163	incomplete	*Bacillus* phage vB_BtS_BMBtp3	7	85.89
BGSC 4C1 prophage-5	36565	3567743	3604308	incomplete	Uncultured *Caudovirales* phage clone 9AX_2	1	82.71
BGSC 4C1 prophage-6	60880	4918921	4979801	incomplete	*Bacillus* phage vB_BtS_BMBtp15	30	96.3

BGSC 4C1 prophage-7	48593	5015923	5064516	complete	Uncultured *Caudovirales* phage clone 3F_9	18	86.75
MYBT18246 prophage-1	108818	333660	442478	complete	*Bacillus* phage vB_BtS_BMBtp16	20	86.29
MYBT18246 prophage-2	40601	631339	671940	incomplete	*Bacillus* phage BMBtp1	44	87.99
MYBT18246 prophage-3	31128	1251048	1282176	incomplete	*Bacillus* phage vB_BtS_BMBtp16	45	82.19
MYBT18246 prophage-4	14607	1343100	1357707	incomplete			
MYBT18246 prophage-5	15817	2455294	2471111	incomplete	*Bacillus* phage vB_BtS_BMBtp16	8	79.59
MYBT18246 prophage-6	28205	2902864	2931069	incomplete			
MYBT18246 prophage-7	63137	3560474	3623611	complete	*Bacillus* phage phIS3501	53	86.06
MYBT18246 prophage-8	74614	3633583	3708197	incomplete	*Bacillus* phage vB_BtS_BMBtp3	2	73.35
MYBT18246 prophage-9	54884	3744292	3799176	complete	*Bacillus* phage phIS3501	40	84.62
MYBT18246 prophage-10	42522	4484401	4526923	complete	Phage Wrath	7	80.38
MYBT18246 prophage-11	45663	5167291	5212954	complete	*Bacillus* phage BMBtp1	6	85.72
MYBT18246 prophage-12	59081	5235229	5294310	incomplete	Uncultured *Caudovirales* phage clone 3F_8	15	85.44
MYBT18246 prophage-13	57012	5384072	5441084	incomplete	*Bacillus* phage vB_BtS_BMBtp16	28	90.16
MYBT18246 prophage-14	35344	5509224	5544568	incomplete	*Bacillus* phage vB_BtS_BMBtp15	53	92.36
MYBT18246 prophage-15	7414	5813321	5820735	incomplete			
KNU-07 prophage-1	54045	1891772	1945817	incomplete	Uncultured *Caudovirales* phage clone 3F_8	20	84.42
KNU-07 prophage-2	38028	2019945	2057973	incomplete	*Bacillus* phage vB_BtS_BMBtp15	1	85
KNU-07 prophage-3	65599	3122355	3187954	incomplete	Uncultured *Caudovirales* phage clone 3F_8	16	84.6
Bt18247 prophage-1	106811	355506	462317	complete	*Bacillus* phage vB_BtS_BMBtp16	21	91.85
Bt18247 prophage-2	61612	784775	846387	complete	*Bacillus* phage BtCS33	50	91.28
Bt18247 prophage-3	46692	1162202	1208894	incomplete	*Bacillus* phage BMBtp1	15	88.43
Bt18247 prophage-4	12819	1411920	1424739	incomplete			
Bt18247 prophage-5	38658	1458087	1496745	incomplete			
Bt18247 prophage-6	13591	2092474	2106065	incomplete	*Bacillus* phage proCM3	35	97.79
Bt18247 prophage-7	51557	2664475	2716032	complete	Uncultured *Caudovirales* phage clone 3F_8	12	85.02
Bt18247 prophage-8	9308	2822205	2831513	incomplete			
Bt18247 prophage-9	67680	5195693	5263373	complete	*Bacillus* phage vB_BtS_BMBtp13	17	82.97
L-7601 prophage-1	34370	1141815	1176185	incomplete	Uncultured *Caudovirales* phage clone 3F_9	47	87.8
L-7601 prophage-2	12845	1231511	1244356	incomplete			
L-7601 prophage-3	55922	1396925	1452847	complete	*Bacillus* phage vB_BtS_BMBtp13	30	94.13
L-7601 prophage-4	65230	1746035	1811265	incomplete			
L-7601 prophage-5	14486	1944173	1958659	incomplete			
L-7601 prophage-6	74011	2017660	2091671	incomplete	*Bacillus* phage BtiUFT6.51-F /*Bacillus* phage phi4I1/*Bacillus* phage BtCS33	7	84.17
L-7601 prophage-7	46734	3069777	3116511	complete	*Bacillus* phage Carmel_SA	52	91.98

L-7601 prophage-8	42078	4635391	4677469	incomplete	*Bacillus* phage vB_BtS_BMBtp15	56	95.4
L-7601 prophage-9	42341	5187539	5229880	incomplete	*Bacillus* phage phi4J1	24	90.76
L-7601 prophage-10	38852	5383654	5422506	incomplete	*Bacillus* phage phi4J1	34	90.78
YGd22-03 prophage-1	49309	1524341	1573650	complete	*Bacillus* phage vB_BtS_BMBtp15	1	75.35
YGd22-03 prophage-2	22838	3059922	3082760	incomplete	*Bacillus* phage vB_BtS_BMBtp15	2	84.1
YGd22-03 prophage-3	42711	3621642	3664353	complete	*Bacillus* phage phIS3501	89	92.98
YGd22-03 prophage-4	38151	4263649	4301800	incomplete	*Bacillus* phage BMBtp1	42	90.45
YGd22-03 prophage-5	54563	5324359	5378922	incomplete	*Bacillus* phage PfEFR-5/*Bacillus* phage PfEFR-4	33	90.66
SCG04-02 prophage-1	22264	1343873	1366137	complete	*Bacillus* phage phiCM3	86	89.17
SCG04-02 prophage-2	11782	2434847	2446629	incomplete	Uncultured *Caudovirales* phage clone 9AX_2	7	78.92
BM-BT15426 prophage-1	15410	3327264	3342674	incomplete	*Bacillus* phage vB_BtS_BMBtp14	6	80.89
BM-BT15426 prophage-2	45372	3630946	3676318	incomplete	*Bacillus* phage PfEFR-5/*Bacillus* phage PfEFR-4	1	91.32
c25 prophage-1	30780	1268968	1299748	incomplete	*Bacillus* phage vB_BtS_BMBtp15	2	84.1
c25 prophage-2	55392	2317959	2373351	complete	*Bacillus* phage phiCM3	38	89.14
c25 prophage-3	16785	2547478	2564263	incomplete	*Bacillus* phage phiCM3	8	83.19
c25 prophage-4	61248	2801418	2862666	complete	*Bacillus* phage vB_BtS_BMBtp16	3	81.88
ATCC 10792-2 prophage-1	39110	611529	650639	incomplete	*Bacillus* phage phiCM3	14	98.09
ATCC 10792-2 prophage-2	20363	1097406	1117769	incomplete			
ATCC 10792-2 prophage-3	13504	3343977	3357481	incomplete			
ATCC 10792-2 prophage-4	64444	3611717	3676161	incomplete	*Bacillus* phage BceA1 /*Staphylococcus* phage SpaA1	10	89.15
ATCC 10792-2 prophage-5	19843	5142697	5162540	incomplete	*Bacillus* phage BMBtp1	2	81.35
ATCC 10792-2 prophage-6	9099	5396438	5405537	incomplete			
ATCC 10792-2 prophage-7	28637	5443822	5472459	complete	Uncultured *Caudovirales* phage clone 3F_8	37	84.42
ATCC 10792-2 prophage-8	19466	5715856	5735322	incomplete	*Bacillus* phage vB_BtS_BMBtp13	8	89.35
ATCC 10792-2 prophage-9	32907	6083676	6116583	incomplete	*Bacillus* phage vB_BtS_BMBtp16	49	92.37
ATCC 10792-2 prophage-10	31384	6197746	6229130	incomplete			
ST7 prophage-1	52194	607713	659907	incomplete	Uncultured *Caudovirales* phage clone 3F_8	20	91.13
ST7 prophage-2	55030	767883	822913	complete	*Bacillus* phage BtiUFT6.51-F	47	88.6
ST7 prophage-3	23096	2662247	2685343	incomplete	*Bacillus* phage vB_BtS_BMBtp14	10	81.81
ST7 prophage-4	44641	2680316	2724957	complete	*Bacillus* phage phi4I1	44	93.17
ST7 prophage-5	23804	2951980	2975784	incomplete	*Bacillus* phage vB_BtS_BMBtp14	5	81.83
ST7 prophage-6	14989	3816675	3831664	incomplete	*Bacillus* phage vB_BtS_BMBtp14	17	81.83
ST7 prophage-7	30962	3926660	3957622	incomplete	*Bacillus* phage BtiUFT6.51-F /*Bacillus* phage phi4I1/*Bacillus* phage BtCS33	6	83.92
ST7 prophage-8	57537	5199517	5257054	complete	Uncultured *Caudovirales* phage clone 3F_8	12	83.34
ATCC 10792-3 prophage-1	66307	1020833	1087140	complete	*Bacillus* phage BVE2	5	89.13
ATCC 10792-3 prophage-2	29304	1304069	1333373	incomplete	*Bacillus* phage BtiUFT6.51-F /*Bacillus* phage phi4I1/*Bacillus* phage BtCS33	6	83.3

ATCC 10792-3 prophage-3	76597	1773960	1850557	complete	*Bacillus* phage BceA1 /*Staphylococcus* phage SpaA1	8	89.15
ATCC 10792-3 prophage-4	12829	2441513	2454342	incomplete			
ATCC 10792-3 prophage-5	58263	2948472	3006735	incomplete	*Bacillus* phage phiCM3	10	98.05
ATCC 10792-3 prophage-6	40127	4224890	4265017	incomplete	*Bacillus* phage vB_BtS_BMBtp16	40	92.68
ATCC 10792-3 prophage-7	48037	5361468	5409505	incomplete	Uncultured *Caudovirales* phage clone 3F_8	22	84.42
XL6 prophage-1	43843	702074	745917	incomplete	*Bacillus* phage PfEFR-5	58	96.77
XL6 prophage-2	29292	2081722	2111014	incomplete			
XL6 prophage-3	42016	3917982	3959998	incomplete	Uncultured *Caudovirales* phage clone 3F_8	12	83.93
XL6 prophage-4	49398	4413904	4463302	complete	Uncultured *Caudovirales* phage clone 9AX_2	39	89.19
T13001 prophage-1	29394	288563	317957	incomplete	*Bacillus* phage vB_BtS_BMBtp16	20	96.98
T13001 prophage-2	22882	3301486	3324368	incomplete	Uncultured *Caudovirales* phage clone 3F_8	29	87.3
T13001 prophage-3	45313	3565109	3610422	complete	*Bacillus* phage PfEFR-4	35	92.33
T13001 prophage-4	45831	5188854	5234685	incomplete	Uncultured *Caudovirales* phage clone 3F_8	4	83.96
T13001 prophage-5	25365	5404806	5430171	incomplete	*Bacillus* phage phi4I1	28	93.55
T13001 prophage-6	19194	5459809	5479003	incomplete	*Bacillus* phage BtiUFT6.51-F /*Bacillus* phage phi4I1/*Bacillus* phage BtCS33	7	85.41
T13001 prophage-7	26666	5701718	5728384	incomplete	*Bacillus* phage phiCM3	3	95.93
T13001 prophage-8	21611	5815466	5837077	incomplete	*Bacillus* phage BMBtp1	5	74.16
T13001 prophage-9	43735	5957707	6001442	incomplete	*Bacillus* phage BMBtp1	35	86.82
T13001 prophage-10	21428	6014165	6035593	incomplete	Uncultured *Caudovirales* phage clone 9AX_2	29	79.81
LM1212 prophage-1	34180	60464	94644	complete	*Bacillus* phage vB_BtS_BMBtp13	35	88.11
LM1212 prophage-2	16087	91453	107540	incomplete	*Bacillus* phage vB_BtS_BMBtp13	7	79.26
LM1212 prophage-3	50130	1041746	1091876	complete	Uncultured *Caudovirales* phage clone 9AX_2	13	82.42
LM1212 prophage-4	44935	2002292	2047227	complete	*Bacillus* phage vB_BtS_BMBtp13	12	82.42
LM1212 prophage-5	47428	3143058	3190486	complete	*Bacillus* phage vB_BtS_BMBtp13	22	90.24
LM1212 prophage-6	67401	4642974	4710375	complete	*Bacillus* phage BVE2	1	79.69
LM1212 prophage-7	20777	4954700	4975477	incomplete	*Bacillus* phage vB_BtS_BMBtp13	3	78.09
LM1212 prophage-8	68368	5318512	5386880	complete	*Bacillus* phage vB_BtS_BMBtp13	18	90.24

Table 3. Putative complete prophages identified in Bt genomes.

Prophages	attR	attL	Integrase	Capsid	Teriminase
	Positions in bacteria chromosomes				
YBT-1518 prophage-5	913910..913922<br>916500..916511	882892..882903<br>888411..888423	899646..900188	909162..910328	903699..9054<br>02
YBT-1518 prophage-10	4284285..4284298<br>4301214..4301225<br>4312543..4312554	4260401..4260412<br>4284285..4284298	4264755..4265477	4280831..4281994	4285529..428<br>7187

YBT-1518 prophage-12	5355469..5355482	5320770..5320783	5355557..5356618	4280831..4281994	5337824..5339518
YBT-1518 prophage-13	5554165..5554176	5518306..5518317	5554361..5555419	5531995..5533167	5536926..5538620
BMB171 prophage-2	2541195..2541206<br>2543969..2543981	2498467..2498479<br>2500548..2500559	2499329..2500438	2521643..2522797	2518121..2519701
YBT-020 prophage-1	443743..443754<br>443793..443804	391947..391958<br>394951..394962	395075..395536	414150..415304	410582..412249
CT-43 prophage-4	1890451..1890464<br>1896349..1896362<br>1910313..1910324	1826113..1826124<br>1837189..1837202	1886954..1887922	1870430..1871554	
CT-43 prophage-6	2639579..2639590<br>2655093..2655104	2588715..2588726<br>2600165..2600176	2598154..2599263	2624360..2625484	2620760..2622436
CT-43 prophage-7	3817025..3817038	3773368..3773381	3801235..3801777	3791939..3793102	3795054..3796712
HD-771 prophage-2	1409459..1409470<br>1409729..1409740<br>1425337..1425348	1359225..1359236<br>1359274..1359285<br>1379258..1379269	1378283..1379242<br>1398889..1399431<br>1411339..1412445	1391295..1392458	1394384..1396057
HD-771 prophage-5	2981152..2981164	2920758..2920770	2960590..2961993	2939486..2940649	2942601..2944256
HD-771 prophage-6	4540385..4540405	4501661..4501681	4501816..4502952	4521807..4522970	4518200..4519855
HD-771 prophage-9	5463643..5463654<br>5487465..5487476<br>5490649..5490660	5442197..5442208<br>5452949..5452960<br>5461773..5461784	5449306..5450475<br>5462482..5463024	5468624..5469778	5465019..5466677
HD-771 prophage-10	5790522..5790533<br>5806181..5806192<br>5806182..5806195	5764272..5764285<br>5768464..5768475<br>5773215..5773226	5764325..5765455	5787041..5788225	5783322..5785049
HD-789 prophage-2	2989118..2989129<br>3000589..3000603	2938754..2938768<br>2939099..2939110	2989579..2990640	2966133..2967299	2969476..2971170
HD-789 prophage-4	3356039..3356050	3328682..3328693	3351848..3352390	3343219..3344382	3346331..3347986
MC28 prophage-4	2969225..2969237<br>2987436..2987447	2934767..2934778<br>2934871..2934883	2961468..2962010	2950761..2951912	2953855..2955522
MC28 prophage-7	3141461..3141474<br>3152012..3152024	3102093..3102105<br>3103507..3103520	3144929..3146059	3123444..3124625	3126585..3128006
MC28 prophage-8	5152570..5152581<br>5157538..5157550	5095369..5095381<br>5103101..5103112	5100279..5101403	3123444..3124625	5124030..5125697
Bt407-1 prophage-2	1845876..1845889<br>1903600..1903613<br>1912085..1912097	1830538..1830550<br>1844439..1844452<br>1845876..1845889	1894213..1895172	1877680..1878804	
Bt407-1 prophage-4	2651952..2651964	2605770..2605782	2623390..2623932	2632838..2633962	2629238..2630914

HD73 prophage-1	296807..296818<br>305981..306050	242738..242807<br>256420..256431	266204..266746	272654..273817	269043..270701
HD73 prophage-2	414574..414585<br>423437..423448<br>437867..437880	376296..376307<br>390634..390645<br>431465..431478	436671..437768	414609..415781	409345..411060
HD73 prophage-3	796996..797007<br>816110..816121<br>818234..818245	760542..760553<br>765971..765982<br>770770..770781	781325..781867	789815..790981	785943..787637
HD73 prophage-4	1337784..1337797	1284495..1284508	1284517..1285920	1306566..1307750	1301488..1303188
HD73 prophage-6	2190068..2190081<br>2190091..2190102<br>2192701..2192713	2138013..2138025<br>2142904..2142915<br>2153151..2153164	2139007..2140098	2160346..2161512	2156475..2158169
HD73 prophage-7	5063056..5063067<br>5078785..5078796	5021812..5021823<br>5028484..5028495	5066377..5067438	5043964..5045130	5047307..5049001
HD73 prophage-8	5274992..5275004	5232007..5232019	5275062..5276198	5255144..5256310	5258224..5259909
IS5056 prophage-4	1897175..1897188<br>1911537..1911550<br>1920478..1920489	1843938..1843949<br>1845350..1845363<br>1904710..1904723	1893687..1894646	1877154..1878278	
IS5056 prophage-6	2645742..2645753<br>2661256..2661267	2373786..2373802<br>2606326..2606337	2604315..2605424	2630522..2631646	2626922..2628598
IS5056 prophage-7	3812229..3812240<br>3820819..3820832	3776967..3776980<br>3800822..3800833	3804979..3805521	3795683..3796846	3798798..3800456
YBT-1520 prophage-1	314093..314105<br>330925..330936<br>333657..333668	243519..243530<br>258484..258495<br>273411..273423	252896..254074	319716..320879	316105..317763
YBT-1520 prophage-2	782681..782693<br>806943..806954<br>813280..813291<br>815404..815415	756854..756865<br>762282..762293<br>765614..765626<br>782793..782804	769460..770557<br>770554..772563	790540..791706	786669..788363
YBT-1520 prophage-3	1323891..1323902	1285256..1285267	1285311..1286714	1305777..1306961	1300711..1302399
YBT-1520 prophage-6	5023953..5023964<br>5042389..5042401	4963649..4963661<br>4963922..4963933	5024616..5025677	5002202..5003368	5005545..5007239
HD-29 prophage-1	204784..204795<br>208753..208765	156843..156855<br>167365..167376	177148..177690	183598..184761	179987..181645
HD-29 prophage-2	896651..896663<br>897587..897598	843002..843013<br>861002..861014	850346..851446	873103..873945	868492..869766
HD-29 prophage-5	5017166..5017178	4946878..4946890	4999387..5000448	4981848..4983218	4984988..4986712
HD-29 prophage-7	5243034..5243046	5200050..5200062	5243089..5244240	5223185..5224351	5226265..5227950

HD-1 prophage-1	279829..279840<br>315798..315809<br>321656..321669<br>324765..324815	258214..258264<br>266129..266140<br>280529..280542<br>284807..284818	267849..269027	275801..277063	278227..279819
HD-1 prophage-2	429557..429568<br>436463..436475	400116..400128<br>412937..412948	414683..415807	438746..439918	433482..435197
YBT1520-2 prophage-1	68940..68951<br>85269..85282<br>93216..93234	19909..19927<br>38803..38816<br>50310..50321	77614..78792	69578..70840	66822..68462
YBT1520-2 prophage-3	973273..973284<br>973545..973557	894807..894819<br>913244..913255	911531..912592	933840..935006	929969..931663
YBT1520-2 prophage-6	5200300..5200311<br>5202525..5202536<br>5215692..5215705	5153713..5153726<br>5157824..5157835<br>5183607..5183618	5204432..5205532	5182436..5183602	5185780..5187474
HD-1011 prophage-2	2941834..2941846<br>2960325..2960336<br>2962760..2962773	2910951..2910964<br>2914690..2914701<br>2924364..2924376	2913048..2914154	2938135..2939307	2934264..2935958
HD1002 prophage-1	100422..100434	47815..47827	63811..64353	71819..72982	68215..69870
HD1002 prophage-4	483298..483310<br>484339..484350	417583..417594<br>426879..426891	425248..426309	448589..449755	444718..446412
BGSC 4AA1 prophage-2	1223299..1223311	1165897..1165909	1198970..1199890	1189102..1190286	1183874..1185574
BGSC 4AA1 prophage-6	3956573..3956584	3902354..3902365	3939345..3939887	3929306..3930457	3932358..3934025
BGSC 4AA1 prophage-7	4639815..4639826	4597672..4597683	4598017..4599471	4618279..4619448	4621384..4623081
BGSC 4AA1 prophage-8	5245207..5245219<br>5262888..5262901	5189813..5189826<br>5197140..5197152	5234168..5234710	5225606..5226772	5228949..5230643
YC-10 prophage-1	617782..617793<br>626584..626595<br>634251..634262	579100..579111<br>579404..579415<br>592304..592315	629400..630491	607988..609154	611331..613025
YC-10 prophage-3	1996308..1996319<br>1998533..1998544<br>2011700..2011713	1949726..1949739<br>1953837..1953848<br>1979619..1979630	2000440..2001540	1978448..1979614	1981791..1983485
YC-10 prophage-4	2374688..2374699<br>2376389..2376400<br>2382908..2382921	2331105..2331116<br>2335257..2335268<br>2376562..2376575	2331696..2332793<br>2332790..2334799<br>2377897..2379021	2353682..2354854	2358403..2360118
YC-10 prophage-5	2511281..2511292<br>2540981..2540994<br>2542673..2542691	2466727..2466745<br>2470895..2470906<br>2515557..2515570	2527072..2528250	2493897..2495060	2516280..2517920
YC-10 prophage-6	3199768..3199788	3157229..3157249	3157384..3158520	3177271..3178437	3173672..3175357

YC-10 prophage-7	3429547..3429558<br>3429819..3429831	3353688..3353700<br>3372124..3372135	3370411..3371472	3392719..3393885	3388848..3390542
HS18-1 prophage-4	2546971..2546984	2496265..2496278	2509207..2509749	2518570..2519742	2514699..2516393
HD521 prophage-1	2010879..2010890<br>2022161..2022172	1988162..1988173<br>1994315..1994326	1996061..1997197	2024093..2025079	2019188..2020594
HD521 prophage-3	2356518..2356530<br>2363907..2363918<br>2376056..2376068	2287686..2287698<br>2287764..2287775<br>2288331..2288343	2365944..2366486	2326820..2727944	
YWC2-8 prophage-3	939232..939243<br>939504..939516	863370..863382<br>881807..881818	880094..881155	902403..903569	898532..900226
YWC2-8 prophage-4	3801771..3801782<br>3810573..3810584<br>3818241..3818252	3763087..3763098<br>3763391..3763402<br>3776293..3776304	3813390..3814481	3791977..3793143	3795320..3797014
YWC2-8 prophage-6	5180356..5180367<br>5182581..5182592<br>5195748..5195761	5133773..5133786<br>5137884..5137895<br>5163666..5163677	5184488..5185588	5162495..5163661	5165838..5167532
YWC2-8 prophage-7	5558748..5558759<br>5560449..5560460<br>5566968..5566981	5515164..5515175<br>5519316..5519327<br>5560622..5560635	5561957..5563081	5537741..5538913	5542462..5544177
Pasteur Institute Standard Strain prophage-1	369052..369064	321101..321113	321260..322615	338289..339650	334706..336430
Pasteur Institute Standard Strain prophage-2	609712..609725<br>618038..618051	573162..573175<br>588028..588041	575183..576244	596241..597377	592633..594303
Pasteur Institute Standard Strain prophage-4	1380371..1380382<br>1391180..1391191<br>1394230..1394242<br>1394307..1394320	1295431..1295444<br>1336823..1337014<br>1343537..1343548<br>1357163..1357174	1300976..1302397	1364774..1365952	
Pasteur Institute Standard Strain prophage-5	1915522..1915533<br>1969787..1969798<br>1981784..1981795<br>1981867..1981880	1913253..1913264<br>1918912..1918925<br>1922300..1922311<br>1927102..1927113	1913277..1914242	1942335..1943498	1938724.. 1940382
Pasteur Institute Standard	2095009..2095020<br>2128352..2128365<br>2138311..2138322	2086890..2086901<br>2095228..2095239<br>2105794..2105807	2093643..2094734	2115089..2116255	2111218..2112912

Strain prophage-6					
Pasteur Institute Standard Strain prophage-11	4700897..4700908	4662921..4662932	4700934..4702367	4678924..4679862	4683198..4684418
Pasteur Institute Standard Strain prophage-12	5148153..5148164<br>5150378..5150389<br>5161903..5161916	5100297..5100310<br>5105708..5105719<br>5131451..5131462	5152285..5153385	5130280..5131446	5133623..5135317
Pasteur Institute Standard Strain prophage-14	5550502..5550514<br>5563195..5563208	5512767..5512780<br>5521081..5521093	5553846..5554903	5533860..5535023	5536943..5538598
Bt185 prophage-1	1824972..1824983	1760889..1760900	1825152..1826624	1799465..1800589	
Bt185 prophage-2	2532463..2532476<br>2542020..2542031	2479645..2479656<br>2492171..2492184	2488358..2489467	2513207..2514370	2509661..2511280
Bt185 prophage-3	4284811..4284822	4253115..4253126	4285120..4286511	4265978..4367288	4270719..4272443
HD12 prophage-1	358392..358403<br>358601..358612<br>396888..396899	328204..328215<br>338708..338719<br>342317..342328	340463..341587	346700..347050	360996..362651
HD12 prophage-5	2140940..2140951<br>2140942..2140953	2084122..2084133<br>2088271..2088282	2085115..2086206	2108285..2109451	2102738..2104432
HD12 prophage-9	5139169..5139180	5097838..5097849	5139343..5140404	5129847..5130728	5123018..5124742
HD12 prophage-10	5344066..5344078<br>5361747..5361760	5284393..5284406<br>5291720..5291732	5333027..5333569	5322789..5323955	5327808..5329502
HD12 prophage-11	5452147..5452158	5399779..5399790	5452385..5453737	5430595..5431968	5435560..5437284
Bt407-2 prophage-4	3627284..3627295<br>3646975..3646986<br>3650527..3650539	3724298..3724310<br>3729256..3729267<br>3734313..3734324	3636235..3637782	3658530..3659693	3654956..3656578
HD1 prophage-2	747338..747350<br>763989..764004<br>777927..777938	802952..802963<br>808145..808160<br>823471..823483	778432..778974	786370..787536	782499..784193
HD1 prophage-3	3647841..3647852<br>3648145..3648156<br>3649518..3649530	3684130..3684142<br>3695326..3695337<br>3702994..3703005	3698143..3699234	3676731..3677897	3680074..3681768

HD1 prophage-5	5028288..5028299<br>5052884..5052895<br>5053663..5053674<br>5056259..5056271	5068911..5068923<br>5072587..5072598<br>5080312..5080323<br>5080455..5080466	5056359..5056901	5047246..5048412	5050589..5052283
HD1 prophage-7	5535541..5535559<br>5535775..5535787<br>5539709..5539720	5580097..5580108<br>5595212..5595224<br>5611489..5611507	5569782..5570324	5587852..5589114	5585096..5586736
BGSC 4Y1 prophage-3	3400388..3400399<br>3401398..3401409	3440042..3440053<br>3453046..3453057	3425526..3425972	3417239..3418390	3420291..3421958
BGSC 4Y1 prophage-4	3722547..3722559	3753412..3753424	3734294..3734836	3744015..3745187	3740159..3741838
BGSC 4Y1 prophage-6	4052653..4052664<br>4061278..4061289	4098214..4098225<br>4100468..4100479	4056990..4058186	4078734..4079915	4075080..4076768
T01001 prophage-9	6162406..6162417	6199297..6199308	6162585..6163127	6171260..6172423	6167686..6169308
BGSC 4AJ1 prophage-8	6380454..6380465<br>6385086..6385099	6426968..6426981<br>6428322..6428333	6396051..6396593	6404620..6405792	6400764..6402443
ATCC 10792-1 prophage-5	2436315..2436326<br>2447765..2447776	2487180..2487191<br>2502694..2502705	2462512..2463054	2471960..2473084	2468360..2470036
BGSC 4BA1 prophage-2	1603487..1603498<br>1604897..1604908<br>1618862..1618873	1642131..1642142<br>1650352..1650363<br>1650400..1650411	1603769..1604875	1628856..1630028	1625000..1626679
BGSC 4BD1 prophage-3	3543477..3543489	3574687..3574699	3543596..3544138	3553623..3554786	3550073..3551674
BGSC 4CC1 prophage-2	2381307..2381319<br>2393259..2393270	2427969..2427980<br>2431717..2431729	2383162..2384271	2405987..2407159	2402131..2403810
IBL 200 prophage-4	6106610..6106621	6155589..6155600	6155925..6157082	6135856..6137013	6132379..6133956
IBL 4222 prophage-8	5981134..5981146<br>5985516..5985527	6024967..6024978<br>6026961..6026973	5981849..5983396	6003997..6005160	6000447..6002048
T04001 prophage-5	3422790..3422802	3452701..3452713	280839..281966	3450358..3451521	3446813..3448432
Bc601 prophage-1	248980..249030<br>256895..256906<br>271295..271308<br>275573..275584	270595..270606<br>315961..315972<br>321819..321832<br>324928..324978	250533..250976	266567..267829	268993..270585
Bc601 prophage-2	730098..730109<br>749525..749536<br>751622..751633	775771..775782<br>783765..783776<br>784232..784243	772671..773630	759369..760535	755498..757192
Bc601 prophage-4	2107525..2107537<br>2112416..2112427<br>2120490..2120502<br>2124158..2124171	2122907..2122919<br>2161070..2161083<br>2161093..2161104<br>2163703..2163715	2121919..2122458	2131352..2132518	2127481..2129175

Bc601 prophage-5	4985834..4985846<br>4986107..4986118	5041831..5041842<br>5060267..5060279	5042494..5043555	5021781..5022947	5025124..5026818
Bc601 prophage-6	5210826..5210838	5255381..5255393	44611..45762	5235534..5236700	5238614..5240299
BGSC 4C1 prophage-1	653512..653525	692091..692104	653577..654737	673542..674705	669936..671591
BGSC 4C1 prophage-2	1025186..1025205	1065883..1065902	1025253..1026410	1046828..1047991	1024277..1025233
BGSC 4C1 prophage-7	5015923..5015937	5059625..5059639	5059693..5061048	5055761..5056690	5043126..5044781
MYBT18246 prophage-1	333660..333671<br>343902..343914	404585..404601<br>436267..436279<br>442478..442489	340653..341720	396888..397238	362656..364335
MYBT18246 prophage-7	3560474..3560485<br>3560706..3560717	3618338..3618349<br>3623611..3623622	3605111..3605653	3595314..3596486	3598663..3600357
MYBT18246 prophage-9	3749224..3749235<br>3749477..3749488	3775523..3775534<br>3799176..3799187	3772645..3773187	3777999..3778805	3766831..3768525
MYBT18246 prophage-10	4484401..4484413	4525846..4525858	4525862..4526923	4502334..4503455	4505366..4507033
MYBT18246 prophage-11	5167291..5167303<br>5169443..5169454	5203231..5203242<br>5212954..5212966	5205034..5206095	5171646..5172752	5185209..5185574
Bt18247 prophage-1	355506..355517<br>365762..365773<br>367470..367481<br>378247..378263<br>397188..397199	421815..421826<br>424764..424780<br>432711..432722<br>449284..449295<br>462317..462328	364102..365169	429065..430195	425475..427142
Bt18247 prophage-2	784775..784786<br>796219..796230	844743..844754<br>846387..846398	811500..812042	820040..821212	816169..817863
Bt18247 prophage-7	2664475..2664490<br>2683675..2683686	2715998..2716009<br>2716032..2716047	9603..10481	2701394..2702548	2701393..2702547
Bt18247 prophage-9	5195693..5195704<br>5205339..5205350	5246362..5246373<br>5263373..5263384	5236280..5236822	5225519..5226676	5228595..5230211
L-7601 prophage-3	1396925..1396937<br>1397146..1397158<br>1412615..1412626	1434071..1434082<br>1447531..1447543<br>1452847..1452859	1409067..1410176	1434095..1435258	1430484..1432166
L-7601 prophage-7	3069777..3069788	3115319..3115330	3115450..3116511	3108757..3109638	3098908..3100605
YGd22-03 prophage-1	1524341..1524365	1573650..1573674	1524617..1525753	1548240..1549265	1543767..1545044
YGd22-03 prophage-3	3621642..3621656<br>621703..3621715	3657270..3657282<br>3664353..3664367	3663235..3664296	3640560..3641732	3643908..3645602
SCG04-02 prophage-1	1343873..1343884	1365109..1365120	1344064..1344606	1352988..1354160	1349117..1350811

c25 prophage-2	2317959..2317970<br>2333450..2333462	2358353..2358365<br>2373351..2373362	26483..27025	2335487..2336659	2338836..2340530
c25 prophage-4	2801418..2801430<br>2802066..2802078<br>2807753..2807764	2838948..2838959<br>2851305..2851317<br>2862666..2862678	2857599..2858735	2830849..2831835	2835347..2836624
ATCC 10792-2 prophage-7	5448585..5448597	5472459..5472471	5448642..5449184	5457317..5458480	5453743..5455365
ST7 prophage-2	767883..767894<br>773469..773480<br>776594..776605	815363..815374<br>820788..820799<br>822913..822924	788522..789064	796779..797951	792908..794602
ST7 prophage-4	2680316..2680327<br>2684120..2684132	2712368..2712380<br>2724957..2724968	2681643..2682185	2694190..2695362	2690320..2692014
ST7 prophage-8	5199517..5199528<br>5202453..5202464<br>5205024..5205035	5235816..5235827<br>5236650..5236661<br>5257054..5257065	5242221..5243282	5222258..5223409	5225350..5227005
ATCC 10792-3 prophage-1	1020833..1020844<br>1020879..1020892	1075712..1075725<br>1087140..1087151	1060451..1060993	1050423..1051547	1053471..1055147
ATCC 10792-3 prophage-3	1773960..1773973<br>1777469..1777482<br>1778449..1778460	1814557..1814568<br>1836595..1836608<br>1850557..1850570	1775070..1775897	1805338..1806582	
XL6 prophage-4	4413904..4413915<br>4428086..4428097	4463041..4463052<br>4463302..4463313	4417658..4418767	4442082..4443254	4438211..4439905
T13001 prophage-3	3565109..3565123	3610422..3610436	3573860..3574591	3586499..3587680	3582959..3584533
LM1212 prophage-1	60464..60476	91242..91254	91445..91987	83382..84533	86470..88086
LM1212 prophage-3	1041746..1041757	1090286..1090297	1090374..1091435	1067728..1068900	1071077..1072771
LM1212 prophage-4	2002292..2002303<br>2005844..2005856	2005841..2005852<br>2047227..2047239	2004038..2005162	2026772..2027914	2021635..2023245
LM1212 prophage-5	3143058..3143069<br>3151845..3151864	3171400..3171419<br>3190486..3190497	3162748..3163290	3171432..3172595	3166248..3167933
LM1212 prophage-6	4645921..4647384	4710375..4710388	4645921..4647384	4670083..4671207	
LM1212 prophage-8	5318512..5318523<br>5336898..5336910<br>5370300..5370313	5360398..5360410<br>5370297..5370308<br>5386880..5386893	5370860..5371951	5349876..5351039	5352968..5354650

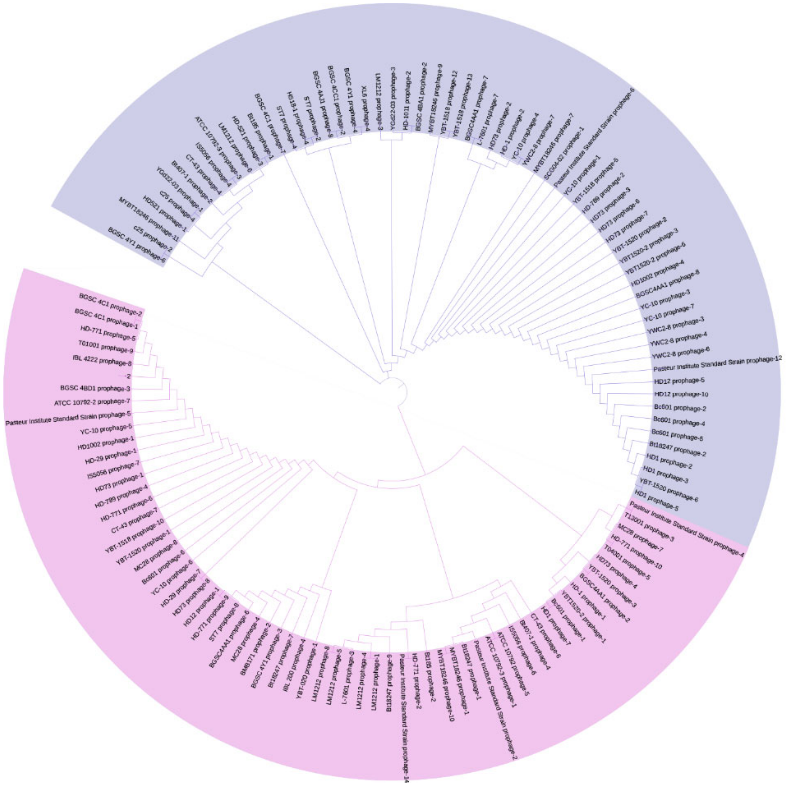

Figure 1. Phylogenetic analysis of Bt prophages by using the major capsid proteins. The amino acid sequences of major capsid proteins from 135 Bt prophages were collected and used for phylogenetic tree construction. The two clades of the phylogenetic tree are indicated in different colors.

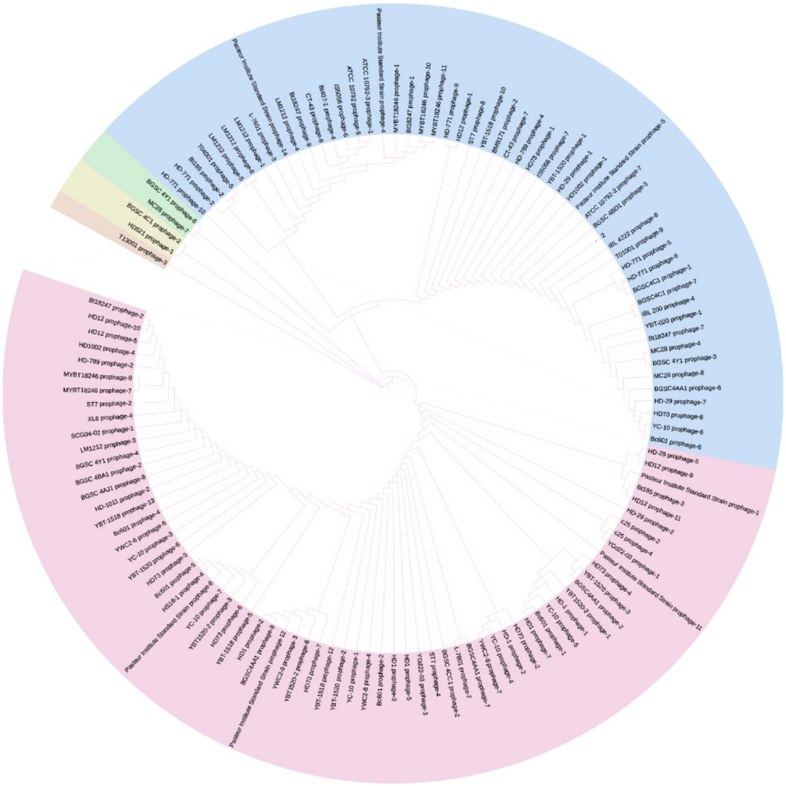

Figure 2. Phylogenetic analysis of Bt prophages by using the terminase large subunit proteins. The amino acid sequences of terminase large subunit from 127 Bt prophages were collected and used for phylogenetic tree construction. The two clades of the phylogenetic tree are indicated in different colors.

Table 4. *Bacillus* phages used for pairwise comparison. The order of phage genomes used for comparison were listed as the rule in this table.

Phage	Accession Number	Genome length	Gene Number
0305phi8-36	NC_009760.1	218948 nt	246
vB_BanS-Tsamsa	NC_023007.1	168876 nt	272
AvesoBmore	NC_028887.1	167431 nt	301
BigBertha	NC_022769.1	165238 nt	291
Phrodo	NC_031100.1	164443 nt	288
Spock	NC_022763.1	164297 nt	283
Zuko	NC_031116.1	163345 nt	294
Nigalana	NC_031037.1	163041 nt	302
Troll	NC_022088.2	163019 nt	289
Riley	NC_024788.1	162816 nt	290
B4	NC_018863.1	162596 nt	277
TsarBomba	NC_028890.1	162486 nt	247
DirtyBetty	NC_031054.1	162415 nt	302
Nemo	NC_031070.1	162375 nt	301
SageFayge	NC_031027.1	162359 nt	300

Belinda	NC_031024.1	162308 nt	295
Eyuki	NC_028944.1	162252 nt	300
Kida	NC_031012.1	162151 nt	304
NotTheCreek	NC_031049.1	161929 nt	296
DIGNKC	NC_031006.1	161552 nt	291
SalinJah	NC_031034.1	161140 nt	292
CAM003	NC_024216.1	160541 nt	287
Bobb	NC_024792.1	160281 nt	247
Hoody T	NC_024205.1	159837 nt	270
Evoli	NC_024207.1	159656 nt	294
BPS10C	NC_023501.1	159590 nt	271
JBP901	NC_027352.1	159492 nt	201
BCP8-2	NC_027355.1	159071 nt	220
Megatron	NC_024211.1	158750 nt	290
vB_BceM_Bc431v3	NC_020873.1	158621 nt	238
BPS13	NC_018857.1	158305 nt	268
Hakuna	NC_024213.1	158100 nt	294
Deep Blue	NC_031056.1	157501 nt	226
W.Ph.	NC_016563.1	156897 nt	274
BCP78	NC_018860.1	156176 nt	227
Bastille	NC_018856.1	153962 nt	273
Bcp1	NC_024137.1	152778 nt	229
Shanette	NC_028983.1	138877 nt	220
CP-51	NC_025423.1	138658 nt	221
JL	NC_028982.1	137918 nt	218
BCD7	NC_019515.1	93839 nt	140
250	NC_029024.1	56505 nt	54
IEBH	NC_011167.1	53104 nt	86
vB_BtS_BMBtp3	NC_028748.2	51366 nt	76
Waukesha92	NC_025424.1	45648 nt	72
phIS3501	NC_019502.1	44401 nt	51
PfEFR-5	NC_031055.1	43773 nt	68
BtCS33	NC_018085.1	41992 nt	57
phi4J1	NC_029008.1	41486 nt	67
PBC1	NC_017976.1	41164 nt	50
BCJA1c	NC_006557.1	41092 nt	58
WBeta	NC_007734.1	40867 nt	53
phiCM3	NC_023599.1	38772 nt	56

phi4B1	NC_028886.1	38663 nt	56
Fah	NC_007814.1	37974 nt	50
TP21-L	NC_011645.1	37456 nt	56
Gamma	NC_007458.1	37253 nt	53
vB_BtS_BMBtp2	NC_019912.1	36932 nt	53
MG-B1	NC_021336.1	27190 nt	42
Claudi	NC_031015.1	26504 nt	46
Aurora	NC_031121.1	25908 nt	40
GA-1	NC_002649.1	21129 nt	35
VMY22	NC_028789.1	18609 nt	25
Bam35c	NC_005258.1	14935 nt	32
GIL16c	NC_006945.1	14844 nt	31
AP50	NC_011523.1	14398 nt	31
Wip1	NC_022094.1	14319 nt	27

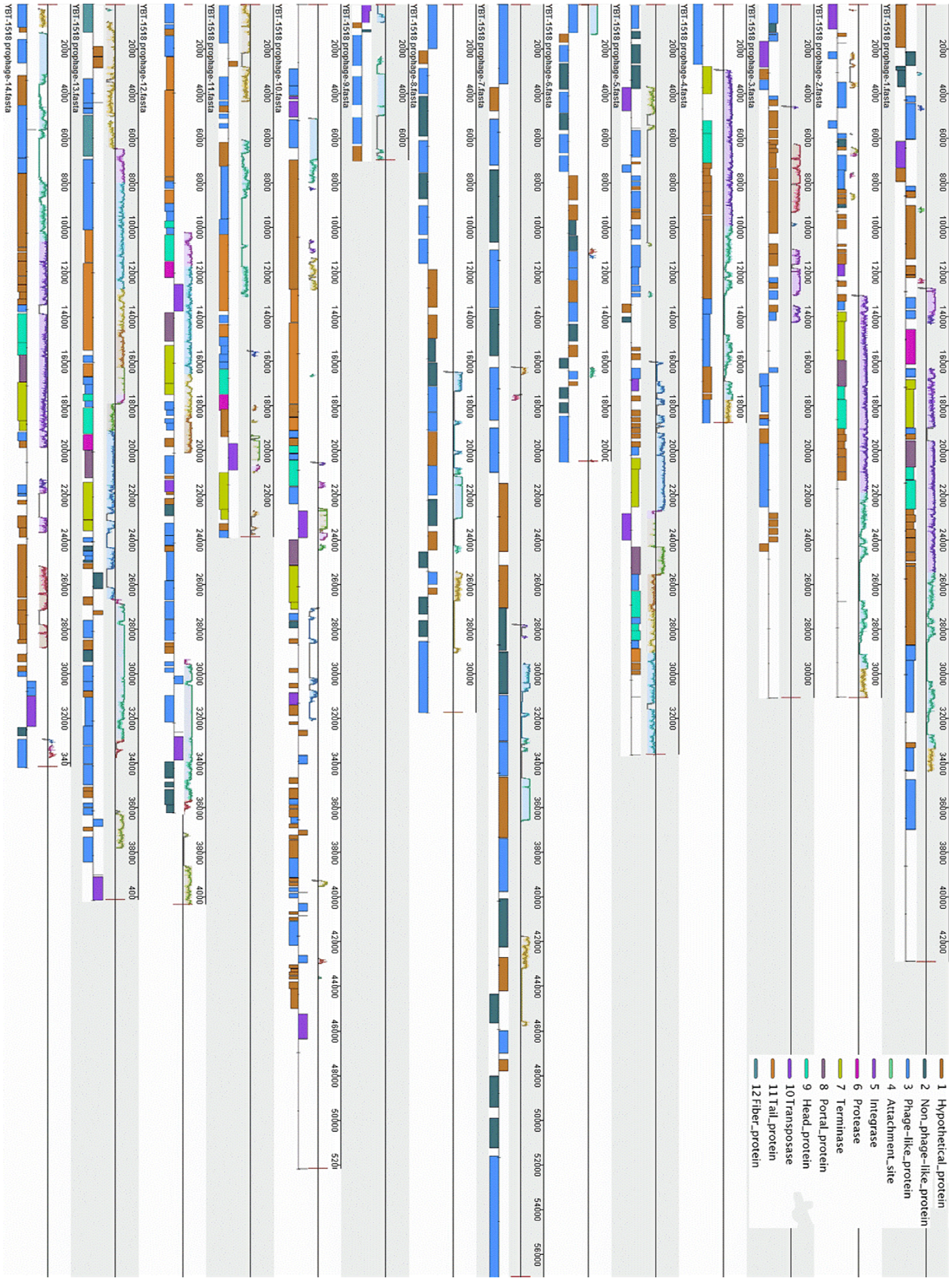

Figure 3. Comparative genomic analysis of prophages from Bt strain YBT-1518. The functions of prophage genes are indicated as different colors and the similarities of the prophage sequences are analyzed by using Mauve.

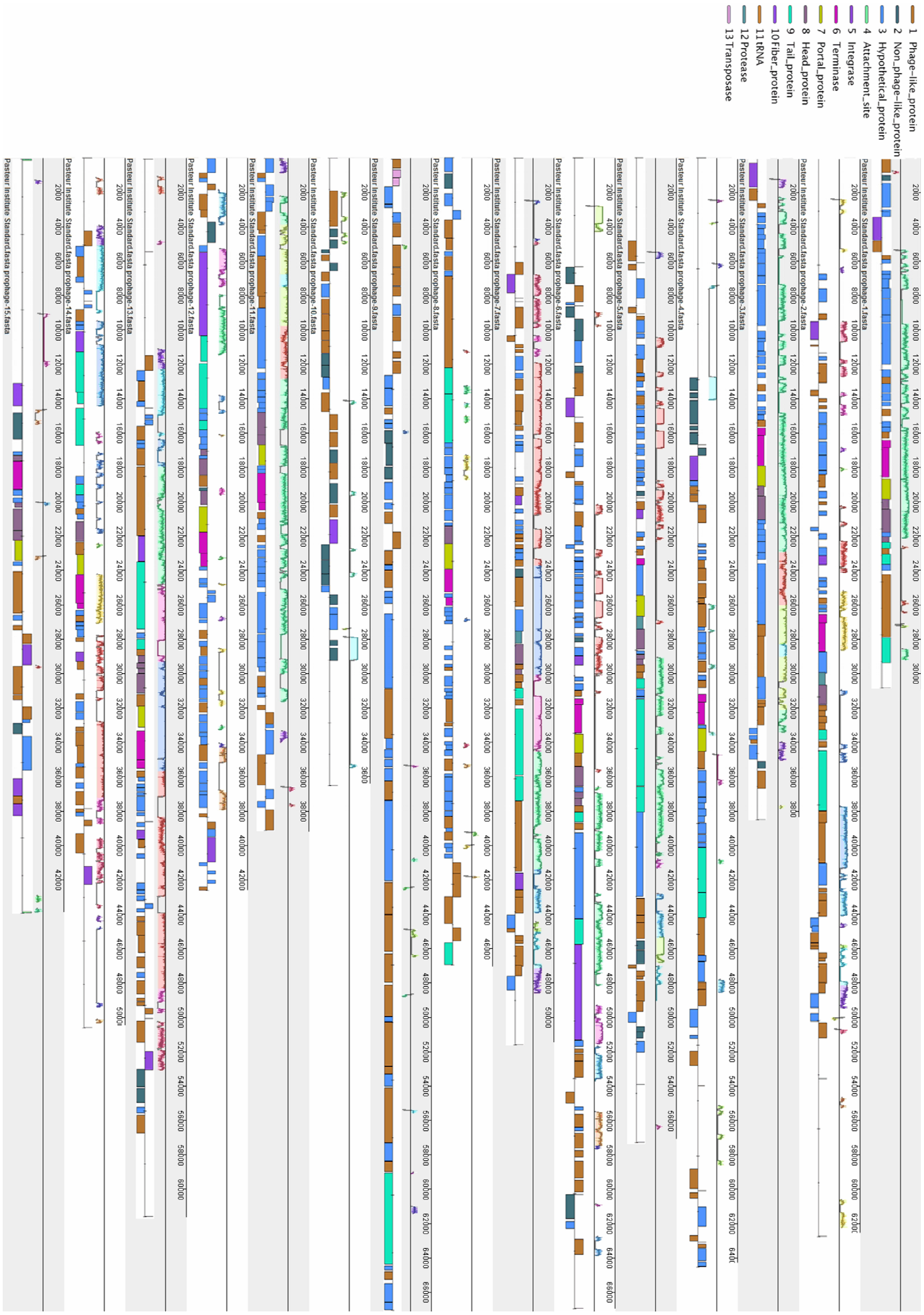

Figure 4. Comparative genomic analysis of prophages from Bt Pasteur Institute Standard strain. The functions of prophage genes are indicated as different colors and the similarities of the prophage sequences are analyzed by using Mauve.

Table 5. Host range of phage induced from Bt strains.

Strain	4C	4A	HD-	HD	4BD1	CT-43	HD-1011	HD-521	HD-73	BMB171

	C 1	J 1	2 9	- 1						
H43	+	+[a]	-	-	-	-	-	+	-	+
4AJ1	+	+	-	-	-	-	-	+	-	-
HD-11	+	+	-	-	-	++	-	+	-	-
HD-201	-	+	-	+	+	-	-	+	-	-
HZ39-04	+	+	-	+	+	-	-	+	-	-
HD-1	+	+	-	-	+	-	-	+	-	-
HD-73	-	+	-	+	-	-	-	+	-	+
HD-541	+	+	-	-	-	-	-	-		-
4BD1	-	+	-	+	-	-	-	+	-	+
YK30-04	+	+	-	+	+	-	-	+	-	-
YGd22-03	-	+	++	-	-	-	-	-	-	-
Scg04-02	-	+	-	-	-	-	-	++	-	-
H33	+	+	-	+	+	-	-	+	-	-
H44	-	+	-	+	+	-	-	+	-	-
HD-868	-	+	-	-	-	-	-	+	-	-
NXP15-04	+	+		+	++	-	-	+		-
H54	+	+	-	+	+	-	-	+	-	-
KK31-01	+	+	-	-	-	-	-	+	-	+
HD-137	+	+	-	+	+	-	-	-	-	-
HD-867	+	+		+	+	-	-	-	-	-
H55	+	+	-	+	+	-	-	+	-	-
HD-521	-	+	-	-	-	-	-	-	-	+
H38	+	+	-	-	+	-	-	+	-	-
HD-866	+	+	-	+	+	-	-	+	-	+
HD-1012	+	+		-	-	-	-	-	-	-
H58	+	+	-	-	+	-	-	+	-	-
HD-7	+	+	-	+	+	-	-	+	-	-
HD-554	-	+	-	-	-	-	-	+	-	+
140	+	+		+	-	-	-	+	+	+
HD-974	+	+	-	+	-	-	-	+	-	+
HD-29	+	++	-	++	-	-	-	+	++	-
H31	+	+	-	+	+	-	-	+	-	-
CT-43	+	+		-	-	-	-	-	-	-
4CC1	-	-	-	-	-	-	-	+	-	-
HD-1011	+	+	-	-	-	-	-	+	-	-
H57	+	+	-	+	-	-	-	-	+	-
H49	+	+	-	-	-	-	-	+	-	+
H39	+	+		-	-	-	-	-	-	-
H16	-	+	-	-	-	-	-	+	-	-
HD-4	+	+	-	+	+	-	-	-	-	-
H23	+	+	-	+	+	-	-	+	-	-
H66	+	+	-	-	-	-	-	+	-	-
BMB171	-	-	-	-	-	+	-	+	-	-

[a] Strains could be infected by induced supernatants and caused lysis zones are indicated as "+", otherwise indicated as "-". The strains used for the purification of the induced phages are indicated as"++".

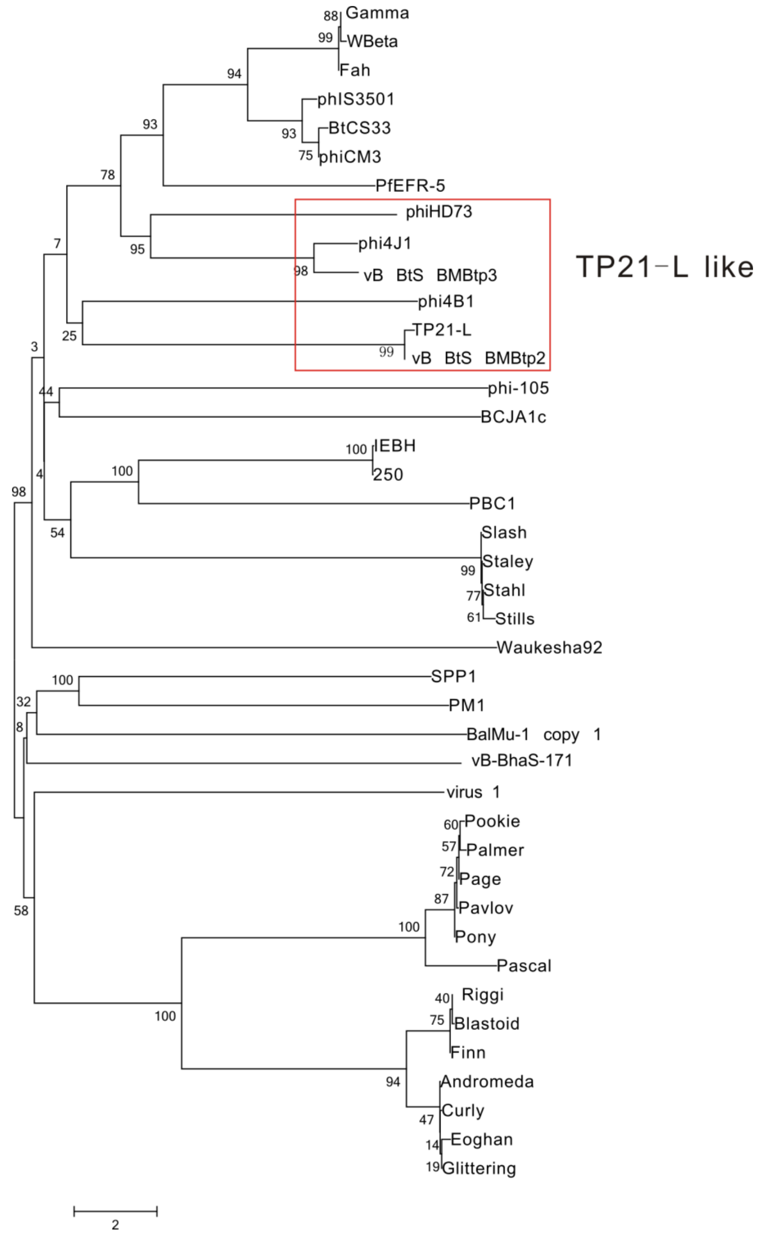

Figure 5. Phylogenetic analysis of 41 *Siphoviridae* family phages. The genome sequences of 40 *Siphoviridae* family phages were collected from GenBank and used for phylogenetic tree construction together with the genome of phage phiHD73.

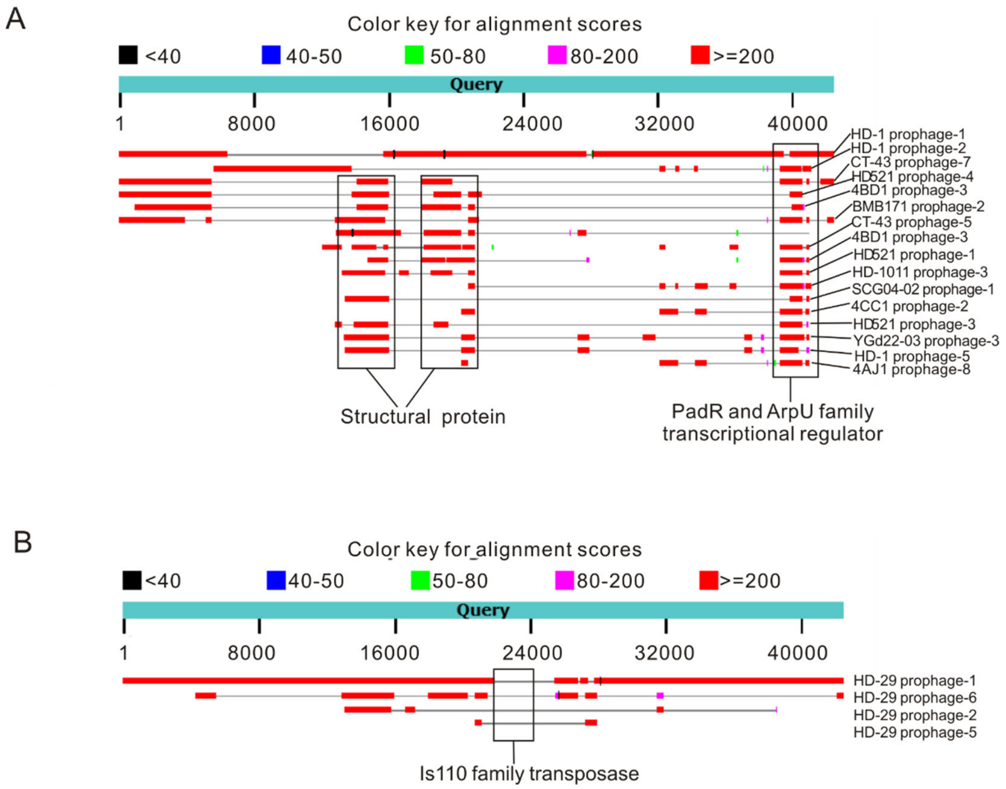

Figure 6. The genome sequences comparison between induced phage phiHD73 and candidate prophages in resistant and susceptive Bt strains by using Blastn. (A) The genome comparison of phiHD73 with prophages in resistant Bt strains. (B) The genome comparison of phiHD73 with prophages in susceptive Bt strain, HD-29, which could be infected by the induced phage phiHD73.

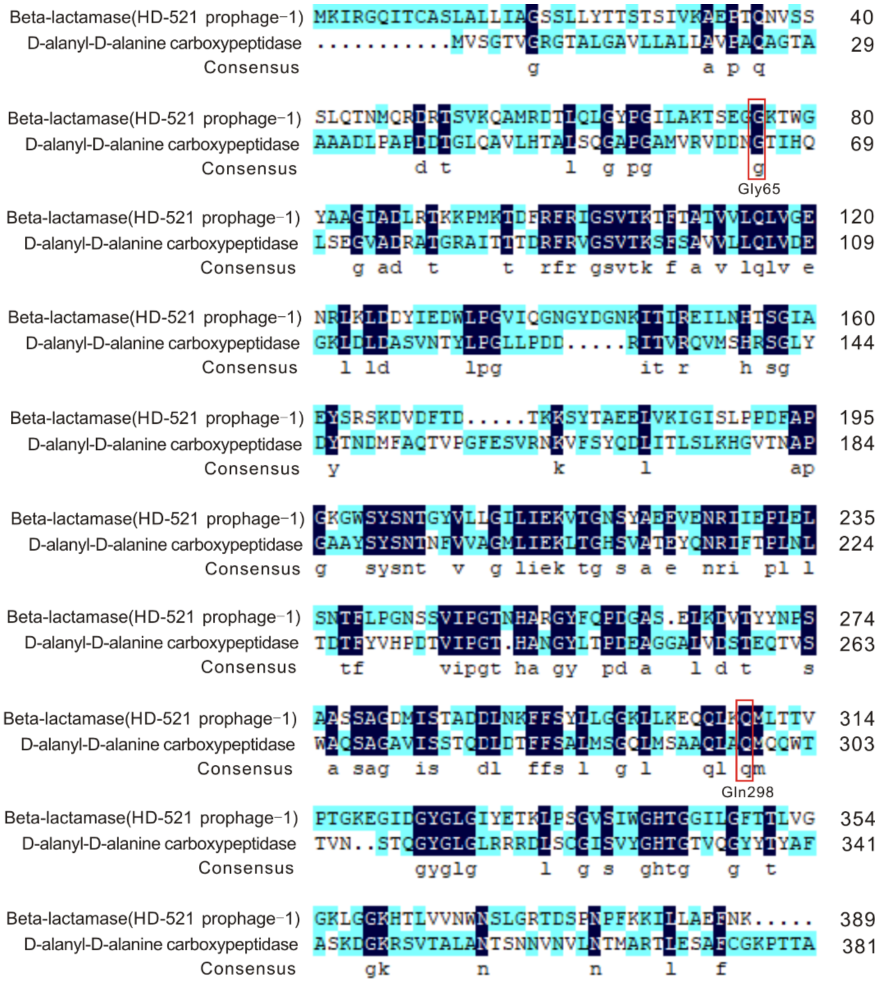

Figure 7. The amino acid sequences alignment of beta-lactamase in HD-521 prophage-1 with the beta-lactamase (PDB database accession number: 1yqs) from *Streptomyces* R61. The catalytic site residues are indicated in red boxes.

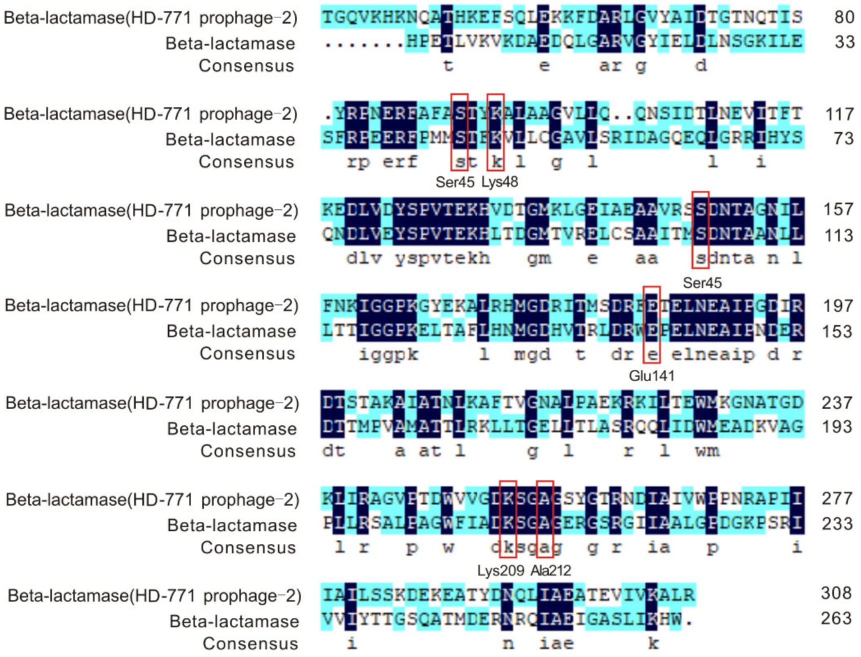

Figure 8. The amino acid sequences alignment of beta-lactamase in HD-771 prophage-2 with the beta-lactamase (PDB database accession number: 1btl) from *Escherichia coli*. The catalytic site residues are indicated in red boxes.

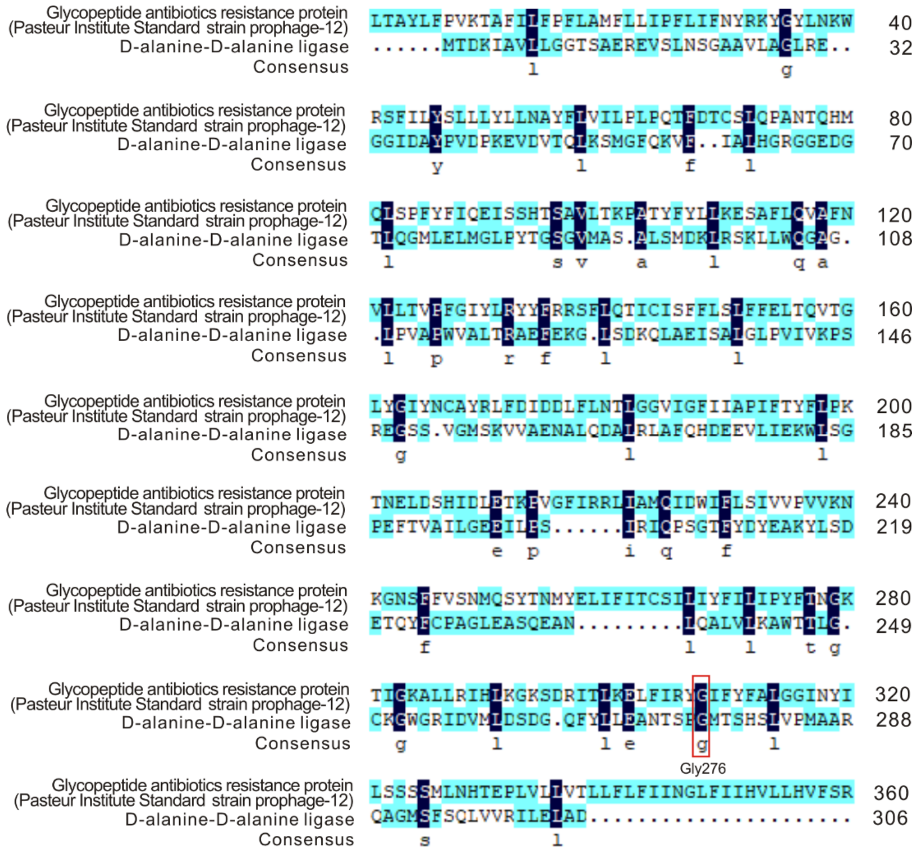

Figure 9. The amino acid sequences alignment of glycopeptide resistance protein in Pasteur Institute Standard strain prophage-12 with the glycopeptide resistance protein (PDB database accession number: 2dln) from *Escherichia coli*. The catalytic site residue is indicated in red box.
